# Digital payments of health workers within vaccination campaigns: a mixed-methods study in Chad

**DOI:** 10.1136/bmjgh-2025-018989

**Published:** 2026-06-24

**Authors:** Esias Ka Nanbe Bedingar, Catherine Wambura, Demilade Osoteku, Antoinette Demian Mbailamen, Adawaye Chatte, Mahamat Fayiz Abakar, Uchenna Igbokwe, Armand Nandoumabe, Briend Donanty Kilone, Muyi Aina, Margaret McConnell

**Affiliations:** 1 Global Health and Population, Harvard University T H Chan School of Public Health, Boston, Massachusetts, USA; 2 Alma, Centre de Recherche en Systèmes de Santé, N’Djamena, Chad; 3 Solina Centre for International Development and Research, Abuja, Nigeria; 4 Vaccination Directorate, Ministry of Public Health and National Solidarity, N’Djamena, Chad; 5 Project Management Unit, Ministry of Public Health and National Solidarity, N’Djamena, Chad; 6 Livestock Research Institute for Development, Ministry of Livestock, N’Djamena, Chad; 7 The University of Alabama at Birmingham School of Public Health, Birmingham, Alabama, USA; 8 National Primary Healthcare Development Agency, Abuja, Nigeria

**Keywords:** Vaccines, Health systems, Health systems evaluation, Qualitative study, Cross-sectional survey

## Abstract

**Introduction:**

Health workers frequently experience inconsistent and inefficient payment processes of their allowances, with potential implications for their motivation, performance and retention and consequently the effective delivery of essential immunisation services. Digitisation of payments has been proposed as a potential improvement to the payment process. This study assesses health workers’ experiences following the digitisation of payments.

**Methods:**

A mixed-methods study was conducted, combining quantitative and qualitative approaches. Quantitative data were collected via a survey of 1510 health workers across 12 provinces (seven intervention, five comparison) between August and October 2023. Additionally, thematic analysis was applied to qualitative data from 57 in-depth interviews with officials and health workers.

**Results:**

Health workers in provinces implementing mobile payments reported higher work motivation (50.7%) and job satisfaction (7.9%) compared with those in comparison provinces (38.67% and 2.7%, respectively). Positive payment experiences were also more frequent in mobile money provinces (14.50% vs 1.36%). Qualitative insights highlighted that timely payments reduced financial stress and enhanced focus on core responsibilities. However, challenges such as technological barriers and the need for tailored training were noted, particularly for workers in remote areas or with lower digital literacy.

**Conclusion:**

Digital payments were linked to higher health worker motivation and satisfaction and perceptions of reliability and reducing administrative burdens. Future research should incorporate longitudinal data and randomised controlled trials to better assess the causal impact of digital payments. Expanding qualitative research to include a broader range of stakeholders will provide deeper insights into the contextual factors influencing the effectiveness of digital payment systems. Addressing these gaps is crucial for refining and scaling digital payment interventions in diverse healthcare settings.

**WHAT IS ALREADY KNOWN ON THIS TOPIC**

- Delayed or inconsistent payments have long undermined the motivation, retention and performance of health workers in fragile and low-resource settings.
- Mobile money has been proposed as a way to improve timeliness, transparency and financial inclusion, but empirical evidence remains limited.

**WHAT THIS STUDY ADDS**

- This mixed-methods study found that digital payments were associated with higher self-motivation and, in some contexts, greater job satisfaction among health workers, particularly where performance-based financing was also implemented.
- While transparency and perceived security improved, technical challenges, transaction costs and uneven access to agents limited perceived benefits, especially for frontline vaccinators.

**HOW THIS STUDY MIGHT AFFECT RESEARCH, PRACTICE OR POLICY**

- Digital payments can enhance workforce motivation if extended beyond managers and paired with complementary reforms.
- Policymakers should invest in infrastructure and user support systems, while future studies should assess causal impacts through longitudinal or experimental designs.

## Introduction

 Vaccination is one of the most effective public health interventions, averting 4–5 million deaths each year by safeguarding against infectious diseases such as measles, polio and diphtheria.^1^ Vaccines significantly reduce morbidity and mortality rates, contribute to disease control and eradication and bolster community health and resilience.^2 3^ Vaccines protect individuals and contribute to public health by achieving herd immunity, thus lowering the incidence of diseases and healthcare costs.^4^ Despite these proven benefits, global immunisation rates remain suboptimal, particularly in low-resource and fragile settings.^5^ Chad, a landlocked country in Central Africa with about 18 million people, has one of the world’s lowest immunisation coverage rates. With a predominantly young population and over 1 million displaced persons and refugees, its health system is under-resourced and unevenly distributed, compounding barriers to care. In countries like Chad, these challenges include logistical difficulties, inadequate infrastructure and limited healthcare resources, which complicate the delivery of immunisation services.^6 7^ Chad has experienced intermittent internal conflict, particularly in the Kanem, Lac and Logone Oriental regions driven by cross-border insurgencies and intercommunal violence.^6 7^ These security challenges disrupt routine vaccination through health worker displacement, supply chain interruptions and community distrust of mobile outreach teams.^6^,^9^ In some areas, checkpoints and roadblocks lengthen supply routes; in others, health posts close temporarily when violence flares.^8 9^ These dynamics compound the structural barriers such as poverty and refugee influxes already undermining immunisation coverage and can limit the reach and continuity of both routine and campaign-based services.

Implementing effective immunisation programmes in low-resource and fragile settings presents significant challenges, particularly those related to the financial and administrative aspects of healthcare delivery. These regions often struggle with inconsistent payment processes and financial insecurity among health workers, which undermine their motivation and capacity to deliver vaccines effectively.^10^ Addressing these systemic issues is essential for enhancing the efficiency and impact of immunisation initiatives in such settings.^11^

A critical yet underexplored element influencing the success of immunisation programmes is the payment process for healthcare workers. Regular and timely payment is crucial for ensuring the financial stability, job satisfaction and motivation of health workers.^12^ Inefficiencies in traditional cash-based payment systems—such as delays, corruption and administrative burdens—can lead to dissatisfaction, absenteeism and reduced workforce productivity.^13^ Although various incentive schemes have been explored to boost health worker performance, fundamental aspects of payment mechanisms—how and when health workers are paid—are less well understood.^14^ Understanding and optimising these payment processes support efforts to enhance the performance and retention of health workers, with the potential to improve immunisation outcomes.^15 16^

Recently, there has been growing interest among governments and multilateral organisations in experimenting with digital payment systems to address these challenges.^17^ Digital payments offer the potential to streamline financial transactions, reduce delays, enhance transparency and minimise the risk of corruption.^18 19^ By providing timely and direct transfers to health workers’ mobile wallets, digital payment systems may alleviate the logistical and administrative burdens associated with cash payments.^20^ They also offer the flexibility to reach remote areas and accommodate the needs of a mobile workforce.^21^ In the context of immunisation programmes, such systems may strengthen worker motivation and efficiency, with potential downstream effects on vaccine delivery.^17 22^

This paper examines the implementation of a digital payment system for vaccination workers in Chad, a country with one of the lowest immunisation rates in the world.^23^ Given the limited evidence on the effects of digitising health worker payments, this mixed-methods study aims to explore how digital payments relate to workers’ motivation, satisfaction and performance in a national vaccine programme in Chad.

To frame this inquiry, we employ expectancy theory (ET), which offers a useful lens for understanding the motivational dynamics influenced by changes in payment systems.^24^ ET is particularly apt for this context because it examines the relationship between effort, performance and rewards, providing insights into how modifications in payment processes can enhance workers’ perceptions of these linkages.^25 26^ Unlike other motivational theories, such as Maslow’s hierarchy of needs or Herzberg’s two-factor theory,^27 28^ which emphasise broader or intrinsic motivational factors, ET focuses on specific, transactional aspects of motivation. This makes it well suited for analysing how concrete changes in payment mechanisms influence health workers’ expectations and their resultant motivation and satisfaction, although it has also been critiqued for assuming rational decision-making and overlooking structural and cultural influences that shape motivation in real-world contexts.^29^,^31^

The introduction of a digital payment system has the potential to reshape the effort-performance-reward relationship by enhancing payment reliability and reducing administrative burdens.^32^ By applying ET, this study aims to deepen understanding of how such reforms influence health workers’ beliefs about effort and reward, their motivation and ultimately their role in improving immunisation outcomes.

## Methods

### Study setting and population

Chad, a landlocked country in Central Africa with a population of about 18 million people, is one of the world’s poorest countries,^33^ with 65% of its population under 25 years of age and a median age of 15.1.^33 34^ The fertility rate is 6.35 births per woman, and many women marry and give birth at young ages.^34 35^ Chad hosts over 1 million displaced people and refugees.^36^ Chad’s healthcare system, composed of district hospitals, health centres and community health posts, is under-resourced and unevenly distributed, impacting immunisation coverage and accessibility.^37 38^ Immunisation programmes, supported by the Ministry of Public Health and Prevention (MOPHP), with support from WHO, UNICEF and Gavi, target diseases like measles, polio, tuberculosis and diphtheria, delivered through campaigns (online supplemental table 1). Despite recent improvements, Chad remains one of the countries with the lowest immunisation rates globally (online supplemental table 2).^6 7^ By 2022, coverage for the third dose of diphtheria, tetanus and pertussis (DTP3), the first dose of measles-containing vaccine and DTP1, had increased to 56%, 60% and 98.8%, respectively, with ongoing efforts to improve cold chain logistics and health infrastructure.^7^

### The Routine Immunization Strengthening in Polio High-Risk Geographies intervention

Challenges in routine immunisation in Chad include leadership, governance, financing, supply chain maintenance and service delivery. Poor financing and weak financial management have driven low immunisation rates.^39^ In 2019, the Aliko Dangote Foundation and the Bill & Melinda Gates Foundation (BMGF) signed a Memorandum of Understanding (MoU) with Chad’s government to address these issues.^40^ Before the intervention, health worker payments followed a traditional cascade cash system, where funds moved from the national treasury through provincial and district offices to health facilities and managers then paid frontline vaccinators in cash. This process was frequently delayed, opaque and vulnerable to leakage, leaving many workers uncertain about when they would be paid. The MoU sought to change this by creating a dedicated basket fund for routine immunisation, introducing annual budget lines and requiring provincial health delegations, districts and facilities to open mobile money payment accounts for direct transfers. Building on this foundation, the Ministry of Public Health implemented a digital payment system using Moov Money to make disbursements more efficient and transparent transactions.^40^,^42^ Moov Money, a mobile money service by Moov Telecom, offers secure and convenient digital financial services, particularly in areas with limited banking infrastructure.^42^ Under this model, the Program Management Unit transfers funds quarterly to Moov Money, which disburses them monthly to managers across various administrative levels—national, provincial, district and health centre levels (online supplemental file 1A). These managers withdraw the funds from mobile money agents and distribute cash to frontline health workers. This makes the system a semidirect model: it accelerates and secures fund flows upstream but frontline workers still ultimately receive cash. Health facility managers, who are often also clinicians due to workforce shortages, play a central role in this model. They oversee daily facility operations, manage staff and coordinate immunisation activities while also handling the withdrawal and redistribution of campaign funds (online supplemental file 1B).

### Study design

**Figure 1:**
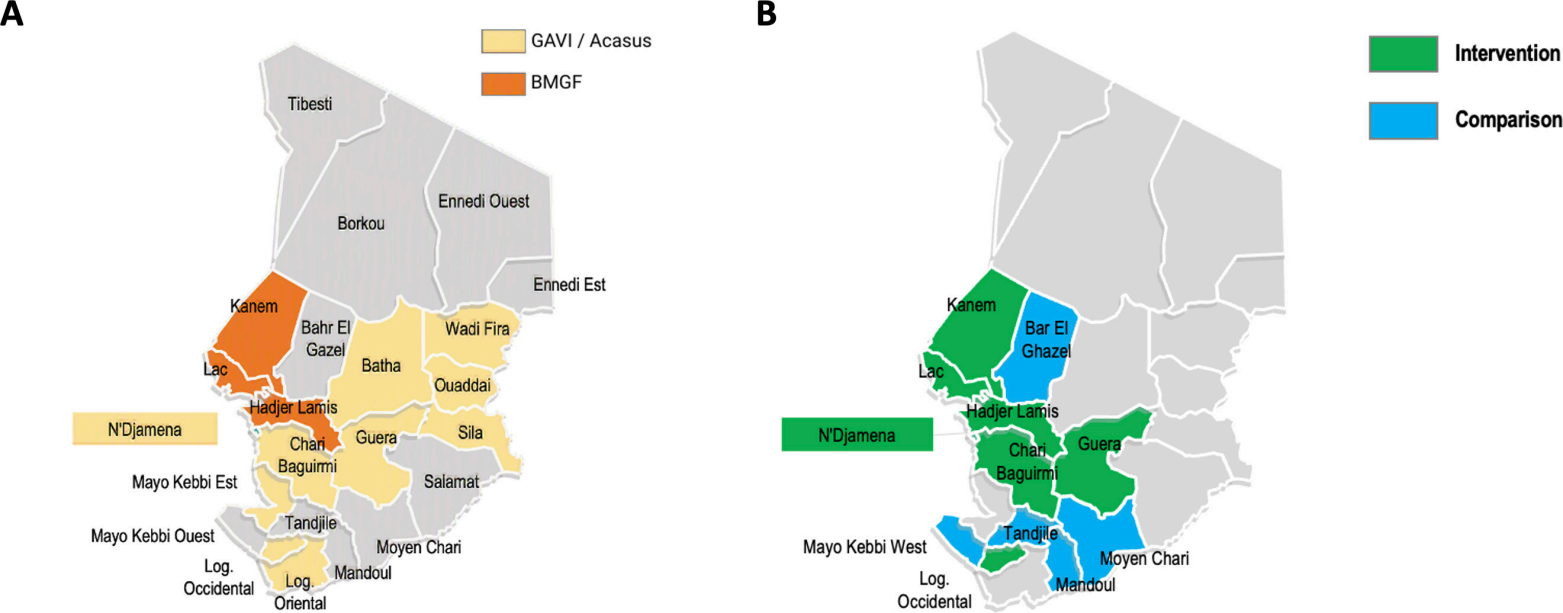
(**A**) RISP intervention provinces distributed between BMGF and Gavi/Acasus. (**B**) Sampled intervention and comparison provinces (green areas indicate intervention provinces, and blue areas indicate comparison provinces). BMGF, Bill & Melinda Gates Foundation; Log, Logone; RISP, Routine Immunization Strengthening in Polio High-Risk Geographies.

To understand health workers’ experiences with the digital payment system, we used a convergent parallel mixed-methods design, collecting and analysing quantitative and qualitative data independently, then merging the results.^43^ A cross-sectional survey measured job motivation, satisfaction and operational efficiency, while semistructured interviews explored experiences in provinces with and without the digital payment system. The study targeted vaccination workers across 12 provinces, with seven provinces using mobile money and five as comparison sites (figure 1). Comparison provinces were chosen based on two criteria: security feasibility (excluding areas with significant instability that would compromise fieldwork) and programmatic alignment, meaning they were provinces where immunisation activities were already being supported by partners such as BMGF, Gavi or Acasus, similar to intervention provinces but without the mobile money component (figure 1).

This study uses ET to examine how digital payment systems impact worker motivation, satisfaction and performance in vaccination services through a mixed-methods approach.^24^ ET helps understand how changes in payment processes affect the relationships between effort, performance and rewards, making it an effective framework for this analysis.^24^

### Overlap with performance-based financing

Our study was conducted between August and October 2023, overlapping with the World Bank’s Health System Strengthening Project (P172504), which began scaling up performance-based financing (PBF) in Chad in 2021.^44^ The project’s objective is to improve the utilisation and quality of essential health services, particularly reproductive, maternal, child and adolescent health and nutrition, by linking facility subsidies to performance. PBF was active in 12 provinces, several of which overlapped with our study sample, including Kanem, Lac, Mandoul, Moyen Chari, Guera and Tandjile.^44^ Because PBF could independently influence worker motivation, payment satisfaction and job satisfaction, we incorporate PBF status into our study models.

### Quantitative data collection and analysis

The quantitative study included 1510 health workers, selected using a multistage sampling method across 12 provinces (figure 1 and online supplemental file 2). The quantitative survey tool included questions on motivation, satisfaction and performance using a 5-point Likert scale (online supplemental file 3). It measured aspects like absenteeism, time allocation for immunisation activities, district meeting attendance and vaccine delivery. Since the digital payment system operates as a semidirect model, the implementation outcomes—including confidence in digital payment systems, frequency of receiving digital payments, frequency of receiving cash payments and experiences of payment delays—focused solely on health facility managers. Data were collected between August and October 2023 using tablets and an Android web-based application: CSEntry (CSPro version 7.7.3), with survey tools in French and Arabic. 24 data collectors received 2-day training, and supervisors reviewed geocoordinates for quality assurance.

Descriptive data were presented as counts with percentages for categorical variables and as means with SDs for continuous variables, in accordance with the Strengthening the Reporting of Observational Studies in Epidemiology (STROBE) guidelines.^45^ Comparisons of health worker characteristics between mobile money intervention and comparison provinces were assessed using χ^2^ tests for categorical variables and Student’s t*-*tests for continuous variables. Distributions of Likert outcomes were presented in full to preserve response variability.^46 47^

Our primary analysis employed ordinary least squares (OLS) regression models to compare outcomes between mobile money implementing and comparison provinces, estimating the average differences in reported levels of motivation and satisfaction. Given the semidirect nature of the payment model, in which managers receive digital transfers while frontline workers continue to be paid in cash, we directly stratified the main analysis by managerial status (facility managers vs non-managers). This approach allowed us to capture heterogeneity in payment flows and responsibilities.

Secondary sensitivity analyses were conducted to assess the robustness of the ordinal findings by dichotomising the Likert outcomes into binary measures, an approach often used to facilitate comparability with prior studies and enhance policy readability, recognising that this approach reduces variability and statistical power.^48^,^51^ We then estimated OLS regressions on these binary outcomes, treating indicators as continuous responses.^52^,^54^ In cut-off 1, only those reporting the highest category ‘very motivated/satisfied’ were coded as 1, with all other responses coded as 0. In cut-off 2, the two highest categories ‘motivated/satisfied’ and ‘very motivated/satisfied’ were combined into one, and the remaining categories coded as 0. All adjusted models included the following covariates: age, sex, education level, contract type, cadre, sufficient facility funding, experience of salary delay and province population estimates. To address the potential confounding introduced by concurrent PBF reforms, we estimated three models: (1) the full sample, including PBF province status as a covariate; (2) re-estimating models restricting the analysis to provinces without PBF; and (3) re-estimating models restricting the analysis to provinces with PBF. Robust SEs were clustered at the province level in all models.

Subgroup analyses were limited to sex-stratified models (female vs male health workers), conducted in line with the Sex and Gender Equity in Research guidelines (online supplemental file 4).^55^ Given the smaller sample size for women, these analyses are considered exploratory. Outcomes were analysed and interpreted within the ET framework (online supplemental table 3).^24^

### Qualitative data collection and analysis

For the qualitative data collection, we used a purposeful sampling strategy to select interviewees who were most knowledgeable and relevant to the study objectives. 24 locally trained data collectors conducted 57 in-depth interviews (IDIs) with officials at national, provincial and district levels using a standardised interview tool (online supplemental file 5). Respondents were selected based on job level (national, provincial and district), funding source in their respective regions (BMGF, Gavi, none) and job function (participants were eligible if they directly supervised or were involved in vaccination). The data collectors approached selected interviewees individually, shared with them the purpose of the interview and asked them whether they were willing to participate. Interviews, conducted in French and Arabic, lasted about 30 min each and were recorded for quality control. Transcriptions took approximately 2–3 hours per interview.

Qualitative data were analysed using best fit framework synthesis, guided by ET.^24 52^ ET posits that motivation is influenced by expectancy, instrumentality and valence.^24^ An initial ET-based framework guided the analysis of 57 IDIs through thematic analysis.^53^ NVivo software (V.14) facilitated coding,^54^ starting with familiarisation (n=12) and developing initial codes on exposure, experience, satisfaction, impacts and barriers to digital payments.^53^ Iterative coding refined the framework, aligning ET principles with the realities of implementing digital payments in Chad’s healthcare system (online supplemental table 4).^24 52^

### Reflexivity

In terms of reflexivity, the team comprised diverse members with varying backgrounds, expertise and experiences, which enriched the research process and provided a balanced perspective. EKNB (first author), a native of Chad and the lead investigator on the ground, brought invaluable local knowledge and cultural sensitivity to the study, particularly regarding the nuances of Chad’s healthcare system and the socioeconomic context. This background helped in navigating local challenges and in building rapport with study participants. The team also included international researchers with expertise in qualitative and quantitative methods, ensuring methodological rigour.

The research was conducted in French and Arabic, the primary languages in Chad, ensuring clear communication with participants. Locally trained interviewers were employed to conduct interviews, which helped in capturing cultural nuances and gaining trust within the community. This approach minimised potential misunderstandings and allowed for more accurate data collection. Despite the team’s efforts to mitigate bias, we acknowledge the possibility of inherent biases influencing the analysis and interpretation of findings. The lead investigator’s dual role as a researcher and policymaker might have influenced his perspectives on the digital payment system, potentially introducing bias. To address this, the team employed triangulation by sharing preliminary findings with stakeholders not directly involved in the study. Feedback from these external reviews was used to refine the analysis and interpretation of the data.

### Ethical considerations

Authorisation for data collection was also provided by the MOPHP in Chad. All data used in this manuscript are part of that approved protocol and were fully deidentified prior to analysis. No new primary data were collected for this analysis.

## Results

### Sample characteristics

There were 1510 health workers, divided into 848 health workers in mobile money implementing and 662 health workers in comparison provinces (table 1). A significant number of health workers held higher education degrees, with 47.3% in comparison provinces vs 56.3% in mobile money provinces. Out of the 1510 health workers, 714 and 796 were facility managers and vaccination workers, respectively. Due to personnel shortage in some provinces, vaccination workers (n=15) were also facility managers. 57 IDIs were conducted with facility managers (out of 714) to investigate the usability, acceptability and effectiveness of the digital payment system in Chad. Among those interviewed, 31 facility managers were from the mobile money implementing provinces and 26 from the comparison provinces. Table 1 summarises the characteristics of health workers in both quantitative and qualitative studies. Onlinesupplemental tables 5  6 provide additional information about sample characteristics.

**Table 1:** Sample characteristics for health workers and health facilities (n=1510)

	Comparison provinces (n*=*662)	Mobile money implementing provinces (n=848)	P value
	Count (%)		
**Quantitative study: health workers**			
Age, mean (SD)	37.81 (8.36)	37.77 (7.02)	0.457
Sex			
Male	574 (86.7)	724 (85.4)	0.461
Female	88 (13.30)	124 (14.6)	
Education			
None	1 (0.15)	13 (1.53)	<0.001
Primary	34 (5.14)	44 (5.19)	
Secondary	314 (47.43)	314 (37.03)	
Higher	313 (47.28)	477 (56.25)	
Contract type			
No contract	332 (50.15)	307 (36.2)	<0.001
Short term	46 (6.95)	41 (4.83)	
Fixed term	134 (20.24)	102 (12.03)	
Permanent	150 (22.66)	398 (46.93)	
Cadre			
Nurse	241 (36.4)	433 (51.06)	<0.001
Midwife	34 (5.14)	49 (5.78)	
Doctor	6 (0.91)	0 (0)	
Manager	150 (22.66)	50 (5.9)	
Others	231 (34.89)	316 (37.26)	
Sufficient funding			
Yes	102 (15.41)	263 (31.01)	<0.001
No	560 (84.59)	585 (68.99)	
**Qualitative study: health workers (managers)**			
Number of provinces	5 (41.67)	7 (58.33)	
Number of districts	28 (54.90)	23 (45.10)	
Number of managers	26 (45.61)	31 (54.39)	
Job function			
EPI manager	0 (0)	5 (16.13)	
Chief medical officer	21 (80.77)	23 (74.19)	
General practitioner	5 (19.23)	3 (9.68)	
Years in current position			
1–5	13 (50.0)	24 (77.42)	
6–10	12 (46.15)	4 (12.90)	
>10	0 (0)	1 (3.23)	
Missing	1 (3.85)	2 (6.45)	

EPI, Expanded Programme on Immunization.

### Implementation outcomes for managers only

Implementation outcomes for managers (n*=*714) highlighted significant differences between comparison and mobile money implementing provinces (online supplemental table 7). Managers in mobile money implementing provinces had higher confidence in digital payment systems (83.7% vs 76.2%). Among health workers receiving digital transfers, 41.2% in mobile money provinces reported receiving two to three payments in the prior 3 months, compared with 29.0% in comparison provinces. Cash payment patterns also differed, with comparison provinces (60.7%) reporting more frequent cash payments than mobile money provinces (22.5%). However, when considering any payment modality (digital or cash), overall payment receipt was lower in mobile money provinces. Approximately 32% of managers in mobile provinces received no payment in the previous 3 months, compared with 7% in comparison provinces. Payment delays also varied meaningfully. Reports of no payment delays were more common in mobile provinces, with 66.3% indicating they never experienced delays in the past 3 months, compared with 26.5% in comparison provinces.

### Quantitative results

#### Descriptive findings: motivation, satisfaction and payment experiences

Motivation, payment satisfaction and overall job satisfaction showed differences between mobile money implementing and comparison provinces (table 2). For work motivation, nearly half of workers in mobile money implementing provinces reported being strongly motivated (50.7%) compared with 38.7% in comparison provinces, while moderate levels of motivation were common in both groups. Reports of low motivation were rare (<2%). For payment satisfaction, 10.7% of workers in mobile money provinces reported being very satisfied vs 2.6% in comparison provinces. Dissatisfaction clustered in comparison provinces: 39.1% were unsatisfied or very unsatisfied, compared with 22.2% in mobile money implementing provinces. Patterns in overall job satisfaction also favoured mobile money provinces, as one in five (20.1%) were very satisfied compared with just 10.0% in comparison provinces, and neutrality was more frequent (9.3% vs 2.6%).

**Table 2:** Distribution of motivation and satisfaction among health workers, by province type

Outcome (5-point Likert scale)	Comparison provinces (n=662)	Mobile money implementing provinces (n=848)	Total (n=1510)
	Count (%)		
Work motivation			
Strongly disagree	3 (0.5)	3 (0.4)	6 (0.4)
Somewhat disagree	12 (1.8)	10 (1.2)	22 (1.5)
Neither agree nor disagree	26 (3.9)	45 (5.3)	71 (4.7)
Somewhat agree	365 (55.1)	360 (42.7)	725 (48.0)
Strongly agree	256 (38.7)	430 (50.7)	686 (45.4)
Payment satisfaction			
Strongly disagree	403 (60.9)	342 (40.3)	745 (49.3)
Somewhat disagree	227 (34.3)	415 (48.9)	642 (42.5)
Neither agree nor disagree	15 (2.3)	29 (3.4)	44 (2.9)
Somewhat agree	12 (1.8)	41 (4.8)	53 (3.5)
Strongly agree	5 (0.8)	21 (2.5)	26 (1.7)
Overall job satisfaction			
Strongly disagree	343 (51.8)	325 (38.3)	668 (44.2)
Somewhat disagree	253 (38.2)	353 (41.6)	606 (40.1)
Neither agree nor disagree	24 (3.6)	30 (3.5)	54 (3.6)
Somewhat agree	24 (3.6)	73 (8.6)	97 (6.4)
Strongly agree	18 (2.7)	67 (7.9)	85 (5.6)

#### Regression analyses of Likert outcomes (OLS)

Regression analyses adjusting for sociodemographic and facility-level characteristics confirmed that mobile money was associated with differences in health worker outcomes, though the direction and magnitude of effects varied across domains and by PBF exposure (table 3). Full multivariable model outputs, including coefficients for all covariates used in table 3, are provided in online supplemental table 8.

**Table 3:** OLS regression models for mobile money intervention effects on health worker outcomes

Outcome	Full sample	Provinces without PBF	Provinces with PBF
Panel A: managers			
Work motivation	0.53*** (0.35 to 0.70)	−0.26 (−0.53 to 0.02)	1.21*** (1.00 to 1.43)
Payment satisfaction	−0.05 (−0.24 to 0.14)	−0.59*** (−0.86 to −0.32)	0.57*** (0.30 to 0.83)
Job satisfaction	0.36*** (0.16 to 0.55)	−0.31* (−0.60 to −0.03)	0.90*** (0.64 to 1.16)
n	714	374	340
Panel B: non-managers			
Work motivation	−0.02 (−0.18 to 0.14)	0.14 (−0.12 to 0.41)	−0.41** (−0.66 to −0.15)
Payment satisfaction	−0.25*** (−0.38 to −0.11)	−0.22 (−0.49 to 0.05)	−0.31** (−0.51 to −0.11)
Job satisfaction	−0.05 (−0.17 to 0.08)	−0.29* (−0.54 to −0.04)	0.01 (−0.17 to 0.20)
n	796	372	424

Coefficients with 95% CIs are presented from OLS regression models. Statistical significance is indicated by asterisks, with *p<0.05, **p<0.01, ***p<0.001. All models were adjusted for potential confounders including age, sex, cadre, education, contract type, location, safety and population size. Results are shown for the full sample, as well as separately for provinces without PBF and provinces with PBF exposure. The outcomes were measured on 5-point Likert scales, with work motivation ranging from 1 (very low) to 5 (very high), and payment and job satisfaction ranging from 1 (very dissatisfied) to 5 (very satisfied). Higher coefficients indicate a relative increase in the outcome score, measured in Likert points, associated with the mobile money intervention. Complete model specifications and coefficient estimate for all confounders are reported in online supplemental table 8.

OLS, ordinary least squares; PBF, performance-based financing.

Among managers (panel A), mobile money was strongly associated with higher work motivation in the full sample, with an average increase of 0.53 points on the 5-point scale (95% CI 0.35 to 0.70; p<0.001). This effect was not significant in non-PBF provinces but was large and positive in PBF provinces (β=1.21, 95% CI 1.00 to 1.43; p<0.001). For payment satisfaction, there was no effect in the full sample (β=−0.05, 95% CI −0.24 to −0.14), but model specification revealed a significant negative association in non-PBF provinces (β=−0.59, 95% CI −0.86 to −0.32; p<0.001) and a positive association in PBF provinces (β=0.57, 95% CI 0.30 to 0.83; p<0.001). Similarly, managers reported higher job satisfaction overall (β=0.36, 95% CI 0.16 to 0.55; p<0.001), though this masked a negative association in non-PBF provinces (β=−0.31, 95% CI −0.60 to −0.03; p<0.05) and a significant association in PBF provinces (β=0.90, 95% CI 0.64 to 1.16; p<0.001).

Among non-managers (panel B), mobile money was not significantly associated with work motivation in the full sample, nor in non-PBF provinces. However, in PBF provinces, non-managers reported significantly lower motivation (β=−0.41, 95% CI −0.66 to −0.15; p<0.01). For payment satisfaction, negative associations were observed across specifications: lower satisfaction in the full sample and in PBF provinces, with a similar but non-significant trend in non-PBF provinces. Finally, job satisfaction showed no overall association, though non-managers in non-PBF provinces reported lower satisfaction (β=−0.29, 95% CI −0.54 to −0.04; p<0.05), while no effect was observed in PBF provinces.

Sex-disaggregated descriptive and regression analyses are shown in onlinesupplemental tables 9  10, respectively.

#### Robustness checks with binary cut-offs

To further assess the robustness of findings, we examined the distribution of motivation and satisfaction outcomes under two alternative binary definitions (online supplemental table 11). Using cut-off 1 (very favourable only), 45.4% of workers were very motivated, 7.1% were very satisfied with payment and 15.7% were very satisfied with their job. Using cut-off 2 (two most favourable categories), 93.4% were motivated, 58.2% were satisfied with payment and 84.9% were satisfied with their job. Regression model OLS on the binary outcomes is shown in onlinesupplemental tables 12  13.

Under the cut-off 1 specification (online supplemental table 12), mobile money was associated with higher adjusted means on the ‘very motivated/satisfied’ indicators among managers in PBF provinces across all three outcomes, including work motivation, payment satisfaction and job satisfaction, consistent with the main analyses for this subgroup. In contrast, associations for managers in non-PBF provinces were small or non-significant, and among non-managers the results were mixed to negative, particularly in PBF provinces. Taken together, the cut-off 1 results indicated that the strongest improvements at the very top category were concentrated among managers where PBF was in place, whereas other groups did not exhibit comparable gains.

Under the cut-off 2 specification (online supplemental table 13), the OLS mean differences were smaller and less consistent. For managers in PBF provinces, the positive patterns seen at cut-off 1 attenuated and, for some outcomes, notably job satisfaction, became negative or null. Among non-managers in non-PBF provinces, a few modest positive mean differences emerged for payment satisfaction, but elsewhere the estimates remained close to zero or negative. Overall, the second cut-off 2 results suggested that when the threshold was broadened to include the second-highest category, the associations weakened, and the direction became less uniform across cadres and settings.

### Qualitative results

Themes identified in the study are presented in table 4. Qualitative findings identified four themes and 14 subthemes, including utilisation outcomes such as exposure, experience and satisfaction, as well as potential impacts and barriers to digital payments.

**Table 4:** Qualitative analysis in themes

Themes	Categories	Codes	Exemplar quotes
**Payment set-up**			
	Utilisation	Use; use only for malaria campaigns	“Digital payments exist in the area where I am responsible for payment for mosquito net distribution.”
	Implementation	Implementation	*“*I participated in the implementation of digital payments for health workers by providing the database of the 24 health centers who activities I supervise.”
	Responsibility of the healthcare managers on payment	Responsible; no one	"Everyone is involved in each stage of the payment because the agent gives his account to his superiors, who then transmit it to us, and we send it to the partners so that they can directly transfer it to the account for the beneficiary. And after that we check with the vaccinators on the ground to see if they have actually received their money. So, everyone is involved.”
**Acceptance of the digital payment system**			
	Job improvement	Improvement; role stayed the same; data transmission	“There’s a clear improvement in the transmission of data from health centers to the district.”
	Readiness of vaccine workers	Not ready for the payment	“In my experience, digital payment is good, the reactions of agents who are not educated say that it is a scam but the others take it very well and the digital payment system is only good.”
	Preference for cash payments	Scam; reliability; speed	“I haven't experimented but cash payment is better compared to this new system.”
**Health system improvements**			
	Efficiency	Stakeholders; regular payments; speed; waiting line	“The number of people has decreased considerably, and the work is going well.*”*
	Transparency	Transparent transactions	“I think the transparency of transactions has changed since the introduction of this system because the beneficiary no longer needs to come to the manager’s office to earn their money. He is just waiting for his mobile payment agent to receive his money.”
	Human resources	Payment traceability; planning	“Digital payment has shortened the journey that our human resources took to collect money.”
	Experience of vaccine workers	Security; motivation; confidence	“The part that changed is the computerized side for vaccination agents who have never had a bank account, it is a plus for them to discover the technological mode.”
**Challenges in the implementation and use of the digital payment system**			
	Slow payments	Speed; health authorities; slow payments	“I have not noticed any speed because for more than two months since the accounts were opened for the mosquito net distribution campaign, nothing has been done.*”*
	Supplementary fees	Availability of withdrawal points; travel costs; withdrawal fees	“Speaking of convenience of transactions the change is not felt as it should be because the system is not yet used. But I can say that this payment system would not be effective for the area because you have to travel a distance of 58 km before arriving at the exchange point.”
	Disagreement	Fund allocation; conflicts; misuse	“Often, we use our money to organize training, room rental, catering and sometimes we do not receive this amount, creating a lot of conflicts with local partners because of late payments and the omission of this amount due to the partners.”
	Technical issues	SIM cards; network issues	“The most common types of issues in payment processing for vaccination workers are: Loss of simcards and renewal difficulties.”

#### Theme 1: payment set-up

##### Utilisation and implementation

Managers in mobile money provinces frequently reported using the digital payment system, though some noted it was initially restricted to specific activities such as mosquito net distribution. One manager explained: *“*Digital payments are currently used in all health centers in my area of responsibility*”* (Intervention, PBF; district level). Another facility manager noted limited use: *“*Digital payments exist in the area where I am responsible for payment for mosquito net distribution*”* (Intervention, non-PBF; district level). Several managers (n=8) described active involvement in setting up the system, contributing to its initial implementation by providing databases of healthcare workers: *“*I participated in the implementation of digital payments for health workers by providing the database of the 24 health centers who activities I supervise*”* (Intervention, PBF; district level).

##### Responsibility

Responsibility for the digital payment system was seen as a collaborative effort among donors, vaccine agents and managers in the mobile money provinces:

Everyone is involved in each stage of the payment because the agent gives his account to his superiors who then transmit it to us, and we send it to the partners so that they can directly transfer it to the account for the beneficiary. And after that we check with the vaccinators on the ground to see if they have received their money. So, everyone is involved. (Intervention, PBF; provincial level)

In contrast, comparison provinces left the responsibility largely to the Ministry of Health and mobile phone companies, as evidenced by: “As far as I know, those responsible for the payment circuit are the governments, the mobile phones [companies], and the vaccinators*”* (Comparison, non-PBF; facility level).

### Theme 2: acceptance of the digital payment system

#### Job improvement

The digital payment system was generally viewed positively for improving job performance by simplifying data transmission and reducing the need for physical travel to collect payments: *“*At district EPI, digital payment makes it easier for me to organize monthly routine vaccination monitoring meetings*”* (Intervention, PBF; district level). Another manager mentioned, *“*There’s a clear improvement in the transmission of data from health centers to the district*”* (Intervention, PBF; district level).

#### Readiness of vaccine workers

There were mixed reactions regarding readiness for digital payments. Less educated workers showed resistance, perceiving digital payments as complex or unreliable: “The reactions of agents who are not educated say that it is a scam, but the others take it very well […]*”* (Intervention, PBF; district level). However, educated workers and those familiar with digital systems adapted better, reflecting the varying valence placed on the new payment system. Others have expressed concern that the new digital payment method would make their job obsolete. This concern stems from a perceived decrease in the need for their specific role of handling payments between the government and the vaccine workers. This sentiment is captured in the quote below:

For me, digital payments have come to replace us because we no longer have enough of a role to play. In addition, the number of people is almost wiped out or eliminated with the arrival of digital payments. It is mainly the managers who manage the money of the activities during the cash payment. Now, we just sign the papers, and the money no longer passes through but leaves the government directly into the agents’ accounts, so we can say that several officials are no longer in the running. We see that the manager becomes an extra without a payroll function, but rather an office worker. This may push us to return to N’Djamena to do other work as we no longer have things to do in the lagoon. (Comparison, non-PBF; district level)

#### Preference for cash payments

Some facility managers from the comparison provinces expressed a preference for cash payments due to their perceived reliability and speed, citing concerns about digital payment efficiency and suggesting improvements were needed before wider implementation: *“*We prefer cash payment which is good and fast*”* (Comparison, non-PBF; district level).

### Theme 3: health system improvements

#### Efficiency

Respondents highlighted significant improvements in payment efficiency with digital payments, reducing delays and administrative burdens, as demonstrated by: *“*The introduction of digital payments has positively increased the speed of payment processing, which is becoming faster and more efficient*”* (Intervention, PBF; district level). They further emphasised that reducing the number of stakeholders involved in the payment process contributed to a more streamlined and efficient system. Finally, managers mentioned that payments to their vaccine workers have become more regular and faster, indicating a positive shift in the overall payment dynamics. They also mentioned that an improvement was seen by eliminating waiting lines for vaccine workers to receive payment, as shown by: *“*The waiting line has changed, directly into the beneficiary’s account without waiting for managers as before*”* (Intervention, PBF; provincial level).

#### Transparency

Digital payments enhanced transparency, reducing complaints and ensuring workers received funds directly: *“*Digital payments allow community relays to receive their money directly in their account*”* (Intervention, non-PBF; facility level).

#### Human resources

The system improved resource management by eliminating the need for physical travel to collect payments. A facility manager shared, “This payment system has changed planning because the travel time to seek liquidity has changed and we are on site to make things happen*”* (Intervention, PBF; provincial level). Another mentioned, *“*The introduction of digital payments has affected the planning and management of health resources in the sense that it provided more financial guarantee to health centers which, before, depended solely on their own resources of the management committees which are very limited*”* (Intervention, PBF; facility level). This improvement positively impacted motivation and satisfaction.

#### Experiences of vaccine workers

Beyond administrative benefits, some respondents from mobile money provinces highlighted the positive effects of the digital payment system on vaccine workers. They reported an increased sense of security among workers due to receiving their payments directly into their accounts, as evidenced by: “Security is guaranteed because everyone has their money with them. It is up to him to protect well*”* (Intervention, PBF; district level). This, in turn, was perceived to have a motivational impact on the workers: *“*This mobile payment system is really a very good way that has made work easier for all our agents*”* (Intervention, PBF; district level).

### Theme 4: challenges in the implementation and use of the digital payment system

#### Slow payments

Despite the benefits of direct digital payments, some respondents reported delays in the digital payment process, causing frustration: *“*It is a system that we accepted with pleasure, but the problem is the slowness*”* (Intervention, non-PBF; facility level). These delays negatively impacted motivation, as workers could not rely on timely payments.

#### Supplementary fees

Additional fees charged by money agents for withdrawals were a significant concern, reducing the effective payment amount, as evidenced by: *“*The fees charged by money agents reduce the actual amount I receive*”* (Comparison, non-PBF; facility level). Another mentioned: *“*The considerable distance to be traveled by vaccinating agents to collect their dues since there is no agent credit or transfer on site. Sometimes this loan officer asks for additional money before paying the vaccinators*”* (Comparison, non-PBF; district level). Additionally, because of the limited availability of digital money agents in certain areas, workers are forced to travel to long distances to withdraw their funds, incurring additional expenses:

Even for an agent to receive his money digitally, he has to travel a certain distance to the city and there, he has to pay for the trip. Let’s assume that he received the transfer of 7500F for the campaign, he must pay the return trip 4000F and the transfer agent will still withdraw a fee of 500F despite the payment being made with the fees. He only has 3000F left. (Comparison, PBF; district level)

Some respondents recommended collaborative efforts among implementors, donors and mobile phone companies to address these fees, as shown by: *“*We want you to send Tigo cash agents to us for payment because the distance for some agents does not help them*”* (Intervention, PBF; provincial level).

#### Disagreement over distribution of money

Conflicts regarding fund allocation for vaccination campaigns and training were reported, often leading to misunderstandings and misuse of funds. These conflicts undermined trust in the payment system and affected motivation by creating uncertainty about reward distribution. One facility manager noted, “I often see that there are disagreements with administrative authorities over the funds allocated for mass vaccination campaign activities, for training and other activities related to payments to health workers*”* (Intervention, non-PBF; facility level). Additionally, facility managers often had to use their own money to organise activities. One facility manager highlighted, *“*Often, we use our money to organize training, room rental, catering and sometimes we do not receive this amount due to the partner*”* (Intervention, non-PBF; district level). These financial delays and omissions caused significant personal financial strain and led to conflicts with local partners, undermining the collaborative efforts essential for successful implementation.

#### Technical issues

Several respondents reported facing technical issues while implementing and using the new digital payment system for their vaccine workers. These challenges included issues like lost SIM cards or forgotten passwords for their mobile money accounts. A facility manager said, *“*The most common types of issues in payment processing for vaccination workers are the loss of sim cards and renewal difficulties*”* (Intervention, non-PBF; district level). Some respondents also reported issues with network connections in their areas, which further delayed access to their money.

### Joint display: integration of quantitative and qualitative results

To enhance integration across strands, we developed a joint display (online supplemental file 6, online supplemental table 14) aligning quantitative outcomes with qualitative themes and exemplar quotes within the ET framework. This display highlights areas of convergence such as higher motivation and job satisfaction among managers in PBF provinces, where qualitative accounts emphasised improved efficiency and transparency and areas of divergence, particularly in payment satisfaction, where new barriers undermined perceived gains. Overall, the integration illustrates that digital payments synergised with PBF to strengthen managerial motivation, while frontline experiences under the semidirect model remained more uneven.

## Discussion

This convergent parallel mixed-methods study from Chad contributes new evidence on how digitising payments affects health worker motivation and satisfaction in a fragile, low-income setting. We found that transitioning from cash to mobile money was associated with higher self-reported work motivation and, in some contexts, greater job satisfaction, particularly among facility managers in provinces already implementing PBF. Non-managers, in other words frontline vaccinators, did not experience consistent benefits, and some reported frustrations with the new payment system. These nuanced outcomes can be interpreted through the lens of the ET, which posits that motivation thrives when workers believe that effort will lead to good performance (expectancy), that performance will be rewarded (instrumentality) and that the reward is valued (valence). In our context, the digital payment intervention appears to have strengthened these linkages for managers, especially where PBF already signalled credible performance rewards, while many vaccinators, particularly outside PBF areas, perceived lower value or certainty, dampening motivational gains.

Our findings align with a growing body of literature from sub-Saharan Africa showing that, under the right conditions, digital payments can improve worker experiences.^17^ Across recent campaigns in multiple African countries, over 80% of frontline workers have preferred digital payment over cash due to a reduced personal risk, convenience and faster disbursements.^17 56^ Timeliness, in particular, is repeatedly linked to better morale and satisfaction; prompt, predictable compensation conveys respect, reduces stress and reinforces commitment to duties.^16 17 56^ This convergence supports the interpretation that reliable digital transfers can strengthen the expectancy and instrumentality components of motivation by making the reward more immediate, traceable and believable. At the same time, our results underscore that digitisation is not a panacea; its impact varies with implementation design, local infrastructure and how directly workers themselves experience the benefits.

A central design feature in Chad was the semidirect disbursement model, where managers at multiple levels received transfers into mobile wallets and then cashed out and paid frontline vaccinators in cash. This arrangement accelerated and secured fund flows upstream but left frontline workers dependent on managers for cash-out. Qualitative interviews revealed how this partial digitisation introduced extra steps and uncertainties for vaccinators, limiting perceived gains in speed or autonomy. The contrast with direct-to-worker designs is notable: when all managers are paid directly, results show more uniformly positive experiences and reduced leakage because funds move straight to the intended recipient and accountability is clearer.^56^ Our findings suggest that paying only managers via mobile money can inadvertently make frontline vaccinators feel bypassed or disadvantaged, lowering satisfaction even when motivation rises modestly. Several vaccinators described mobile money as ‘complex’ or ‘unreliable’ and reported noticing faster payments, echoing experiences elsewhere where digital systems are poorly executed or when cash-out is costly or inconvenient.^57^

Digital literacy and trust moderated these effects. Our qualitative data indicated that less educated workers and those with limited exposure to technology were more likely to view the mobile money system with suspicion, whereas their more digitally savvy colleagues adapted readily and appreciated the innovation. This heterogeneity resonates with regional studies showing that perceived risk, prior experience and trust strongly shape adoption.^58^ For our setting, the implication is practical: rolling out a platform is insufficient without targeted capacity building, including simple training, helplines and peer support tailored to cadres with lower digital familiarity. Without such scaffolding, digitisation risks widening existing hierarchies, with managers accruing more of the benefits. Encouragingly, qualitative accounts indicated that with even modest support, many initially hesitant workers became more comfortable with mobile payments, suggesting that trust building and just-in-time assistance can mitigate early frictions.^56^

Digitisation also reshaped perceived roles along the payment chain. Under cash-based processes, provincial and district managers physically handled funds, a task intertwined with status and responsibility. With mobile payments, funds flowed directly to managers’ SIMs across levels, reducing cash handling and altering routines. Some district-level managers felt their responsibilities had been diminished and a few worried that digital systems might replace them in this function. From an ET lens, such role changes can reduce valence if a valued task disappears without a compensating source of meaning or recognition.^2459^,^62^ This is a predictable change management challenge, where reforms designed to improve transparency by bypassing intermediaries may be experienced as a loss of control.^59 61 62^ Clear communication about new responsibilities, coupled with strengthening supervisory planning and reporting functions, can help maintain manager motivation.^59^,^66^ On balance, removing discretionary cash handling from facility managers likely strengthens accountability and frees time for leadership and supervision. Consistent with this, interviews described administrative efficiencies and greater transparency mirroring efficiency gains observed in other immunisation programmes that digitised payments.^17^

The interplay between digital payments and PBF is a key contribution of this study. Quantitatively, associations between mobile money and higher motivation and job satisfaction were strongest in provinces already implementing PBF, particularly among managers, suggesting a complementary relationship. This is plausible given evidence that well-designed PBF can influence provider motivation when credibly implemented, and that digital payments reliably improve payment timeliness, transparency and auditability in health campaigns, thereby creating conditions that reinforce PBF’s instrumentality signal.^1767^,^74^ We observed the highest motivation and satisfaction among managers in PBF areas, consistent with a synergistic mechanism: prompt, visible disbursements bolster trust that performance will be rewarded, making effort more worthwhile.^75^,^77^ Conversely, in settings without PBF or for cadres not directly benefiting from it, digital reforms sometimes generated disappointment or perceptions of ‘crowding out’, particularly under semidirect implementation that left vaccinators reliant on managers for cash.^78 79^ Reports from other contexts echo these patterns: when digital systems introduce delays, fees or access barriers—such as long distances to agents or liquidity shortages—managers may prefer cash despite digitisation’s theoretical advantages.^57 70 80 81^ Although rigorous causal evidence on digital-PBF interactions remains limited, adjacent literatures support the mechanism we observe: where incentive schemes hinge on credible, timely payment, digital delivery systems can help them perform as intended.^82^,^85^

These findings align with broader multicountry evidence that payment timeliness and completeness are central determinants of health worker motivation and satisfaction. Zhang *et al*^86^ found that salary delays were widespread and consistently associated with lower motivation, reduced job satisfaction and higher absenteeism among primary care workers. This reinforces the interpretation that disruptions in payment systems, regardless of whether payments are delivered digitally or in cash, can undermine workforce morale and performance. In this light, our finding that overall payment receipt in mobile money provinces during rollout likely reflects transitional frictions, including liquidity constraints, staggered onboarding and cash-out barriers, rather than a failure of digital payments per se. Recognising and addressing these early-phase challenges may be essential for maximising the motivational benefits of digital reforms.

### Policy implications

Our findings suggest that digital payments should be tailored to context rather than implemented as one-size-fits-all reform. First, shifting to direct-to-worker transfers is critical to maximise transparency and ensure frontline staff experience the benefits, avoiding the burdens created by semidirect arrangements. Second, transaction costs and access barriers—such as withdrawal fees, long distances to agents and liquidity shortages—must be addressed, for example, through removal or negotiated fee reductions, expanded agent networks or alternative cash-out channels. Third, digital literacy and trust require investment. Simple training, user support mechanisms and clear communication can reduce scepticism and build confidence in mobile money systems. Fourth, integration with PBF (or analogous performance schemes) appears advantageous. Aligning reliable, traceable digital payments with performance incentives helps ensure that timeliness and credibility reinforce broader motivation signals rather than operating in parallel. Finally, change management matters, especially where digitisation alters roles, proactively redefining responsibilities and recognising new supervisory or data functions can sustain manager motivation.

### Limitations

The study has several limitations that should be considered when interpreting the findings. First, as an observational study in a real-world setting, we could only establish associations rather than prove causality. Unmeasured confounders such as differences in leadership, baseline infrastructure or worker characteristics between provinces may have influenced the results. We attempted to control for major factors, including the presence of PBF, but residual confounding cannot be ruled out. Second, the semirandomised rollout of the digital payment intervention means there may have been selection biases. Provinces with better prior performance or resources might have been more likely to implement mobile payments early, potentially overestimating the benefits. Third, our quantitative measures relied on self-reported perceptions of motivation, satisfaction and payment experiences. These subjective outcomes are prone to reporting bias. For example, participants aware of being in a ‘digital’ province might have been more inclined to report improved motivation due to courtesy bias or optimism about a donor-supported innovation. Likewise, those facing technical issues might have been disproportionately negative. We mitigated this by assuring confidentiality and triangulating with qualitative data, but some bias remains possible. Fourth, the qualitative component, while invaluable for explaining the *why* and *how* digital payments shaped worker experiences, was limited in its balance across cadres and settings. Managers were more represented than frontline vaccinators, and perspectives from non-PBF provinces were comparatively fewer. As a result, some frontline or non-PBF-specific experiences may have been under-represented, and the depth of triangulation across groups is narrower than ideal. Finally, our findings are from a specific context and may not generalise to all settings. Countries differ in mobile money ecosystem maturity, network coverage, banking infrastructure and workforce dynamics. Despite these limitations, the study provides timely insights by combining quantitative and qualitative evidence on a relatively under-researched health systems intervention. We believe the lessons learnt can inform digital payment initiatives in similar low-resource and fragile contexts, especially in sub-Saharan Africa.

## Conclusion

This study contributes new evidence on the implementation of digital payment systems for health workers in Chad, a fragile and conflict-affected setting with limited digital infrastructure. Using a mixed-methods design, we found that digital payments were associated with higher self-reported motivation and, in some contexts, greater job satisfaction, particularly when combined with PBF. At the same time, payment satisfaction was lower in mobile money provinces, reflecting persistent challenges such as technological barriers, limited agent access in remote areas, transaction fees and gaps in digital literacy. These findings underscore both the promise and complexity of digital payments: while they improve efficiency, transparency and morale, their impact depends on design features and context. Future research should examine direct-to-worker payment models, explore interactions with existing financing mechanisms and employ causal and longitudinal designs to assess sustainability and broader system effects.

## Supplementary material

10.1136/bmjgh-2025-018989 online supplemental table 1

10.1136/bmjgh-2025-018989 online supplemental table 2

10.1136/bmjgh-2025-018989 online supplemental file 1

10.1136/bmjgh-2025-018989 online supplemental file 2

10.1136/bmjgh-2025-018989 online supplemental file 3

10.1136/bmjgh-2025-018989 online supplemental file 4

10.1136/bmjgh-2025-018989 online supplemental file 5

10.1136/bmjgh-2025-018989 online supplemental table 3

10.1136/bmjgh-2025-018989 online supplemental table 4

10.1136/bmjgh-2025-018989 online supplemental table 5

10.1136/bmjgh-2025-018989 online supplemental table 6

10.1136/bmjgh-2025-018989 online supplemental table 7

10.1136/bmjgh-2025-018989 online supplemental table 8

10.1136/bmjgh-2025-018989 online supplemental table 9

10.1136/bmjgh-2025-018989 online supplemental table 10

10.1136/bmjgh-2025-018989 online supplemental table 11

10.1136/bmjgh-2025-018989 online supplemental table 12

10.1136/bmjgh-2025-018989 online supplemental table 13

10.1136/bmjgh-2025-018989 online supplemental file 6

10.1136/bmjgh-2025-018989 online supplemental table 14

10.1136/bmjgh-2025-018989 online supplemental file 7

## Data Availability

Data are available upon reasonable request. All data relevant to the study are included in the article or uploaded as supplementary information.
