# Supplementary material for: Digital payments of health workers within vaccination campaigns: a mixed-methods study in Chad
Source: BMJ Glob Health. 2026 Jun 24;11(6):e018989. doi: 10.1136/bmjgh-2025-018989 (PMC13295920; doi:10.1136/bmjgh-2025-018989)
Supplement: online supplemental table 1 [file bmjgh-11-6-s001.docx]

**Supplementary table 1:** National immunization schedule for children and pregnant women in Chad^[[1]](#footnote-1)^.

| **Time in point in schedule** | **Vaccine** | **Upper age limit** |
| --- | --- | --- |
| Birth | BCG | Up to 1 year |
| Birth | OPV0 | Up to 15 days after birth |
| 6 weeks | OPV1 | Up to 5 years |
| 6 weeks | Penta 1 | Up to 1 year |
| 10 weeks | OPV2 | Up to 5 years |
| 10 weeks | Penta 2 | At any age (as long as first dose is given by 1 year) |
| 14 weeks | OPV3 | Up to 5 years |
| 14 weeks | Penta 3 | At any age (as long as first dose is given by 1 year) |
| 14 weeks | IPV1 | Up to 1 year |
| 9 months | IPV2 | At any age (as long as first dose is given by 1 year) |
| 9 months | VAR 1 | Up to 5 years |
| 9 months | YF | Up to 59 years |
| 9 months | Men A | Up to 29 years |
| 9 months | VAR 2 | Up to 5 years |
| During pregnancy | Td2+ | During labour |

1. Table shows the schedule of vaccines provided for free by Chad’s national immunization to infants (16 months and under) and pregnant women. The vaccines provided include Bacillus Calmette-Guérin (BCG), oral polio vaccine (OPV), pentavalent vaccine (Penta), inactivated polio vaccine (IPV), varicella vaccine (VAR), yellow fever (YF), meningococcal A vaccine (Men A), tetanus and diphtheria vaccine (Td2+). The pentavalent vaccine contains protection against diphtheria, tetanus, pertussis, Haemophilus Influenzae B and Hepatitis B. It replaced three doses of the diphtheria-tetanus-pertussis (DTP1, DTP2, DTP3). [↑](#footnote-ref-1)
