# Supplementary material for: Digital payments of health workers within vaccination campaigns: a mixed-methods study in Chad
Source: BMJ Glob Health. 2026 Jun 24;11(6):e018989. doi: 10.1136/bmjgh-2025-018989 (PMC13295920; doi:10.1136/bmjgh-2025-018989)
Supplement: online supplemental table 2 [file bmjgh-11-6-s002.docx]

**Supplementary table 2:** Immunization coverage rates for selected provinces and vaccines in 2017^[[1]](#footnote-1)^.

| **Provinces** | **Immunization coverage (%)** | | | | | |
| --- | --- | --- | --- | --- | --- | --- |
|  | **BCG** | **OPV0** | **OPV1** | **Penta 1** | **OPV3** | **Penta 3** |
| Barh El Gazel | 49 | 40 | 69 | 71 | 59 | 63 |
| Chari Baguirmi | 58 | 19 | 93 | 94 | 75 | 76 |
| Guera | 61 | 25 | 70 | 81 | 63 | 73 |
| Hadjer Lamis | 63 | 14 | 59 | 73 | 53 | 65 |
| Kanem | 62 | 17 | 66 | 70 | 56 | 60 |
| Lac | 65 | 15 | 80 | 89 | 71 | 78 |
| Log. Occidental | 65 | 29 | 78 | 91 | 71 | 82 |
| Mandoul | 56 | 24 | 68 | 94 | 58 | 85 |
| MKW | 80 | 29 | 64 | 97 | 55 | 88 |
| Moyen Chari | 74 | 33 | 69 | 88 | 61 | 76 |
| N’Djamena | 83 | 59 | 82 | 89 | 73 | 97 |
| Tandjilé | 66 | 22 | 79 | 99 | 72 | 91 |
| National | 66 | 30 | 71 | 85 | 62 | 76 |

***Data source:*** Enquête nationale de couverture vaccinale, Rapport final – Tome 1, Septembre 2017, Ministère de la Santé Publique et l’Organisation Mondiale de la Santé, N’Djamena, Tchad.

1. The selected provinces are the ones sampled for the study, as presented in Figure 1 of the main manuscript. [↑](#footnote-ref-1)
