# Supplementary material for: Digital payments of health workers within vaccination campaigns: a mixed-methods study in Chad
Source: BMJ Glob Health. 2026 Jun 24;11(6):e018989. doi: 10.1136/bmjgh-2025-018989 (PMC13295920; doi:10.1136/bmjgh-2025-018989)
Supplement: online supplemental file 1 [file bmjgh-11-6-s003.docx]

**Supplementary file 1A:** Schema of the mobile money disbursement system.

**
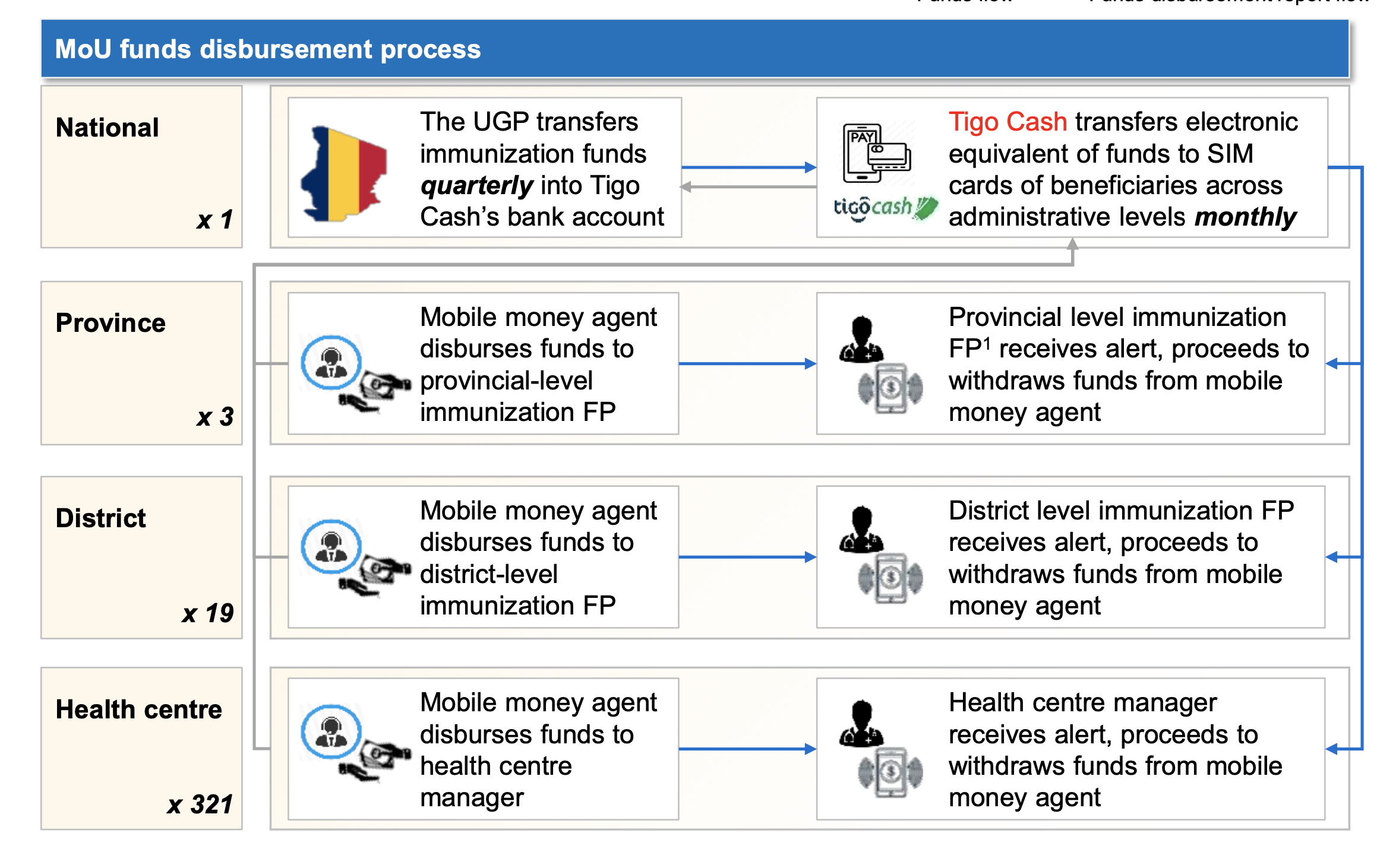
**

***Notes:*** At the national level, the PMU transfers funds quarterly into Moov Money's bank account, which then disburses the electronic equivalent monthly to beneficiaries' SIM cards across various administrative levels. This system operates from the provincial level, where funds are disbursed to immunization focal points, down to district and health center levels. Managers at these levels receive alerts and withdraw funds from mobile money agents, ensuring timely access to resources needed for immunization activities.

**Supplementary file 1B:** Key MoU activities funded by level.

| **Levels** | **Thematic areas** | **Activities** |
| --- | --- | --- |
| National level | Leadership, governance and coordination | Monthly coordination meetings, interministerial committee, bi-annual and annual reviews, operating funds for DLMVSE and UGP. |
|  | Monitoring and evaluation | Formative technical supervision, financial supervision. |
|  | Financial management | Accountant salaries, mobile money transfer cost, printing of management tools. |
|  | Data management | Airtime for uploading data on EPI manager. |
|  | Service delivery | Curative maintenance of the MoU vehicles |
| DSP | Leadership, governance and coordination | Quarterly monitoring meetings |
|  | Monitoring and evaluation | Supervision fueling for vehicles, preventive maintenance fees for vehicles |
| DS | Monitoring and evaluation | Monthly monitoring meetings at the district, fueling for formative supervision |
|  | Service delivery | Mobile strategy fueling for vehicles, preventive maintenance fees for vehicles |
| Health facilities | Monitoring and evaluation | Monthly monitoring meetings |
|  | Service delivery | Fueling for advanced vaccination session (motorbikes), maintenance fees for bikes, boat rental funds for insular places, vaccinators stipend for outreach |
