# Supplementary material for: Digital payments of health workers within vaccination campaigns: a mixed-methods study in Chad
Source: BMJ Glob Health. 2026 Jun 24;11(6):e018989. doi: 10.1136/bmjgh-2025-018989 (PMC13295920; doi:10.1136/bmjgh-2025-018989)
Supplement: online supplemental file 2 [file bmjgh-11-6-s004.docx]

**Supplementary file 2:** Sampling for the cross-sectional survey.

The quantitative study included a total of 1,510 health workers. This comprised 662 workers from comparison provinces and 848 from mobile money implementing provinces. Participants were selected using a multistage sampling method. First, we sampled intervention provinces (7) and selected geographically and administratively comparison provinces (5) for a total of 12 provinces. For accurate representation of the number of health facilities within health districts, we applied a health district weight to the new sample to obtain the number of health facilities to select within districts. Then, we randomly selected the number of health facilities within districts using a random generator. Finally, we interviewed the health facility manager and one randomly selected vaccinator per facility.

The initial sample size was determined on the power calculation for salary delay. Based on health worker surveys in other SSA countries with similar indicators, a 30% salary delay rate was assumed at baseline. Based on the budget and local capacity, the sample size consisted of 1,674 health workers, assuming a cluster size of 2 health workers, 400 clusters in each arm (intervention and comparison), with a significance level of 0.05, power of 80%, and intra-cluster correlation (ICC) of 0.35, during the study period to account for refusals and other factors. The minimum detectable impact was a decrease in salary delay of 6 percentage points or an 20% decrease in salary delay rate.
