# Supplementary material for: Digital payments of health workers within vaccination campaigns: a mixed-methods study in Chad
Source: BMJ Glob Health. 2026 Jun 24;11(6):e018989. doi: 10.1136/bmjgh-2025-018989 (PMC13295920; doi:10.1136/bmjgh-2025-018989)
Supplement: online supplemental file 3 [file bmjgh-11-6-s005.docx]

**Supplementary file 3:** Digital payment survey tool for health center managers and vaccinators.

**General information - Facility**

1. Province, District and Sub-district
2. Level of this facility? Health centre / Clinic / Others
3. Ownership of this facility? Public / Private / Religious
4. Is this facility located in a rural or urban area? Rural / Urban
5. Is this facility located on the mainland / Island?
6. Is this facility located in a security compromised or safe zone?
7. How many health workers work at this facility?
8. Does this facility have an accountant? Yes/No?

**General information - Managers and Vaccinators**

1. Gender: Male / Female
2. How old are you? ______ years
3. What is the highest level of education you have completed? None / Primary school / Secondary school / Tertiary education or above
4. Including yourself, how many people are in your household?
5. Do you live with your family at your work location?
6. How many year(s) and month(s) have you worked as a health worker at this facility?
7. What is your position as a health worker as designated by the Ministry of Health? Manager / Doctor / Nurse / Midwife / [Insert relevant job options] / Other, specify: ____
8. Does the program funding support allow you to cover your needs? Yes/no
9. On average, how much payment (including per diem allowance, transport allowance, meeting and supervision expense, operation expense, etc.) did you receive per month for the vaccination campaign?
10. Did you experience a delay in receiving your payment for the vaccination work? Yes / No
11. What type of employment contract do you have? Permanent and pensionable / Short term contract (less than 6 months) / Fixed term contract (6 months and more) / Casual (no contract) / Volunteer / Other, specify: ______
12. What Province, District and Sub-district did you work in during vaccination campaigns?
13. Overall, how was your payment experience during the vaccine campaign? Very good / Good / Fair / Poor / Very poor

**Digital payments - Health center managers**

1. When have you had a digital payment application installed on your phone? Less than 6 months ago / Between 6 to 12 months ago / More than 12 months ago
2. Did you set it up just for routine immunization / vaccination campaigns? Yes / No
3. Did you run into any issues or challenges when installing the digital payment app? Yes / No
4. How challenging was it? Very difficult / Difficult / Neutral / Easy / Very easy
5. If challenging, did you seek help? Yes / No
6. If challenging, how many times did you have to go back for help? _______times
7. In the last 3 months [or relevant time interval], how many times did you receive digital / cash payments? Digital: _______ times; cash: ______ times
8. Did you experience any delays in receiving these payments? Yes, more than half / Yes, less than half / No
9. Do you know the reasons for these late payments? if yes, please give a brief explanation ____________________________________________
10. [if any digital payments] I’d like to understand how comfortable you are using digital payments. When you use your mobile money account, how often do you ask someone else for help? Always / Sometimes / Rarely / Never
11. Imagine you needed to get cash out of the digital payments. How confident are you that you can get all the cash when you need it? Very confident / Somewhat confident / Not very confident / Not at all confident / Do not know
12. How long does it take you to travel to your nearest mobile money agent? ______minutes / Have not travelled
13. Did you experience any challenges when cashing out the mobile payment? Yes / No / Payment was cashed out by someone else
14. [If yes to the last question] Which of these challenges, did you face when cashing out your payment?
    1. I didn’t know where to find a mobile money agent
    2. I had trouble finding the mobile money agent because they were not at their post
    3. I found a mobile money agent but they didn’t have enough cash to cash out
    4. Line was long
    5. Agent wanted extra money
    6. Forgot PIN / Account blocked
    7. Other (please specify)
    8. I haven’t needed the money yet.
15. Overall, would you prefer the money to be distributed by cash or mobile money for vaccine campaign? Cash / Mobile money / No preference / Not sure
16. What do you think about the functionality of mobile money payments? Functional with some problems/ Functional with no problems/ Not Functional
17. Do you have any suggestions/recommendations for improvement? _______________________________________________________________

**Motivation & satisfaction**

1. [Motivation] To what extent do you agree or disagree with the following statements, with respect to your job as a vaccination campaign worker: These days I feel motivated to work as hard as I can. Strongly agree / Somewhat agree / Neither agree nor disagree / Somewhat disagree / Strongly disagree
2. [Payment satisfaction] How satisfied are you with your payment as a vaccination campaign worker? Very unsatisfied / Unsatisfied / Neutral / Satisfied / Very satisfied
3. [Job satisfaction] How satisfied are you with your job overall as a vaccination campaign worker? Very unsatisfied / Unsatisfied / Neutral / Satisfied / Very satisfied

**Performance**

1. [Absence] Over the last month, were there any days that you were scheduled to work but you were not able to work? yes / no
2. [Time allocation] On a typical day at work, during the campaign, how many hours of this time did you spend on each of the following activities:
   1. Traveling to work
   2. Receiving supervision or training
   3. Attempting to deliver vaccination (locating households, communicating with families, delivering vaccines)
   4. Documenting your work
   5. Eating / drinking / taking break
3. [Managers] In the last 3 months [or relevant time interval], how many district meetings did you attend?
4. [Vaccinators] In the last month [or relevant time interval], how many people did you deliver vaccines to?
