# Supplementary material for: Digital payments of health workers within vaccination campaigns: a mixed-methods study in Chad
Source: BMJ Glob Health. 2026 Jun 24;11(6):e018989. doi: 10.1136/bmjgh-2025-018989 (PMC13295920; doi:10.1136/bmjgh-2025-018989)
Supplement: online supplemental file 4 [file bmjgh-11-6-s006.pdf]

Table 1. SAGER guidelines checklist
Studies with human participants

Section / topic	Item number	Checklist item	Reported on page number
General			
Results	1	The terms sex/gender used appropriately	12, 13
Title			
N/A	2	Title specifies the sex/gender of participants if only one included	N/A
Abstract			
N/A	3a	Abstract specifies the sex/gender of participants if only one included	N/A
	3b	Study population described with sex/gender breakdown*	N/A
Introduction			
	4a	If relevant, previous studies that show presence or lack of sex/gender differences or similarities are cited	N/A
	4b	Mention of whether sex/gender might be an important variant and if differences might be expected	N/A
Sample characteristics	4c	The demographics of the study population with regard to sex/gender (eg, disease prevalence among male/female study participants) are outlined*	12, 13
Methods			
Study setting and population	5a	Method of definition of sex/gender (eg, self-report, genetic testing)	5, 6
Quantitative data collection and analysis	5b	Description of how sex/gender was considered in the design, whether authors ensured adequate representation of male and female study participants, justification of the reasons for any exclusion of male or female participants, or explanation if not considered. Justification of other sex/gender-specific interventions of study designs (eg, mandating contraception for women).* Explicit reporting of the scientific rationale for	10

		contraception requirements and exclusions for pregnancy and lactation should be required*	
Results			
Sample characteristics	6a	Study population description with complete gender/sex breakdown for all categories considered*	12, 13
Regression analyses of Likert outcomes	6b	Where appropriate, data presented disaggregated by sex/gender, and sex/gender differences and similarities are described	16
	6c	Sex- and gender-based analyses reported regardless of outcome (in main paper if pre-specified; otherwise in appendix)*	S7 Table<br>S8 Table
	6d	For clinical trials, adverse event data disaggregated by sex/gender (in main paper if pre-specified; otherwise in appendix)*	N/A
	6e	Patient-reported outcome data disaggregated by sex/gender (in main paper if pre-specified; otherwise in appendix)*	S7 Table<br>S8 Table
	6f	For epidemiological studies, the effects of other exposures on health problems examined for all genders and analysed critically from a gender perspective	N/A
Sample characteristics	6g	Table 1 includes separate rows for male sex/gender, female sex/gender and other categories if collected*	12
Discussion			
	7a	Potential implications of sex/gender on the study results and analyses, including the extent to which the findings can be generalized to all sexes/genders in a population	N/A
	7b	If a sex/gender analysis not done, a rationale is given and implications of the lack of such analysis on the interpretation of the results are discussed	N/A
Adapted from SAGER guidelines. Sex and Gender Equity in Research: rationale for the SAGER guidelines and recommended use. Research Integrity and Peer Review 1, Article number: 2 (2016) https://researchintegrityjournal.biomedcentral.com/articles/10.1186/s41073-016-0007-6.<br>* These points extend beyond the original SAGER table			
