# Supplementary material for: Digital payments of health workers within vaccination campaigns: a mixed-methods study in Chad
Source: BMJ Glob Health. 2026 Jun 24;11(6):e018989. doi: 10.1136/bmjgh-2025-018989 (PMC13295920; doi:10.1136/bmjgh-2025-018989)
Supplement: online supplemental file 5 [file bmjgh-11-6-s007.docx]

**Supplementary file 5:** Digital payment interview tool – national, provincial, district interviewee.

Thank you for taking the time to speak with me. We are very interested in your experience and understanding of how funds for immunization activities are sent to responsible personnel in Chad, and your input will be valuable in evaluating how to improve existing payment systems. Do you have any questions before we begin?

**General information**

- **Position, years in current position**
- **Job function - in general and in the digital payment project (if applicable)**

First, can you tell me the position you currently hold and how long you have been working in this position?

What are your major job responsibilities?

In what ways, if at all, have you been involved with payments for health workers?

[Probe: specifically for vaccination activities, if not mentioned]

Have you been involved in implementing digital payments for health workers in Chad? If so, could you tell me in what ways you have been involved with those activities?

Are digital payments currently being utilized in the geographic region you are responsible for?

**Experience with digital/cash payments**

- **Payment set-up**
- **Routine operations of payments**
- **Barrier to payments**

I would like to take some time to discuss payments to vaccination workers in more detail.

Could you describe, to your best understanding, how money is currently transferred from the national government to an individual vaccination worker for payments related to vaccinations?

[Probe: Who is responsible for each stage of the process?]

(*If digital payments are implemented in respondent’s area of responsibility*)

Now, I’d like to ask some questions about how digital payments have changed how money is transferred.

How has your role changed, if at all, with respect to health worker payments since the introduction of digital payments for vaccination workers?

In your experience, how, if at all, have the number and types of people involved in payments changed since the introduction of digital payments?

[Probe:]

In your experience, how, if at all, has the speed of processing payments changed since the introduction of digital payments?

[Probe:]

(*For all respondents, regardless of whether they are involved with digital payments*)

According to you, what are the types of issues that are most common in processing payments for vaccination workers?

[Probe: How are these issues typically resolved? Are there any other issues that come to mind?]

What should a vaccination worker do if they are having trouble receiving payments?

**Health system efficiency**

Have you observed any changes in the speed of payment processing since the introduction of digital payments? If so, please explain.

[Probe: Which parts of the payment pipeline have seen the biggest changes, if any?]

To what extent do you think that transparency of transactions has changed since the introduction of digital payments, if at all?

[Probe: If changes have been observed, what exactly has changed? For whom has transparency changed?]

To what extent do you think that convenience of transactions has changed since the introduction of digital payments, if at all? How about efficiency of payments? How about the safety of payments?

How, if at all, do you think that the introduction of digital payments has affected the planning and/or management of health resources?

- If yes, please explain how.
- If no, why do you think there hasn’t been much effect? Are there other changes that you think would better improve the planning and management of health resources?

How do you rate the functionality of the mobile money system in general? Functional without issues/Functional with issues/ Not functional

Do you have any suggestions/recommendations for improvement?
