# Supplementary material for: Digital payments of health workers within vaccination campaigns: a mixed-methods study in Chad
Source: BMJ Glob Health. 2026 Jun 24;11(6):e018989. doi: 10.1136/bmjgh-2025-018989 (PMC13295920; doi:10.1136/bmjgh-2025-018989)
Supplement: online supplemental table 3 [file bmjgh-11-6-s008.docx]

**Supplementary table 3:** Conceptual framework linking outcomes to Expectancy Theory (ET) components in the digital payment intervention analysis.

| **ET component** | **Conceptual outcomes** | **Explanation** |
| --- | --- | --- |
| Expectancy | Work motivation | High motivation reflects workers’ belief that their increased effort will lead to better performance. |
|  | Work attendance | Attendance rates indicate workers’ confidence that their presence and effort will result in favorable outcomes. |
|  | Confidence in digital payment systems | High confidence in digital payment systems indicates that workers believe their effort will lead to successful outcomes, as they trust the reliability and efficiency of the payment process. |
| Instrumentality | Payment satisfaction | Satisfaction with payment measures the perception that good performance leads to timely and accurate compensation. |
|  | Positive payment experience | Positive experiences with the payment system indicate the belief that effective performance results in prompt and reliable compensation. |
|  | Reduced payment delays | Fewer payment delays reflect workers’ perception that their performance will be reliably rewarded without undue delays. |
|  | Frequency of digital payments | Regular and frequent digital payments reinforce the belief that good performance is directly linked to consistent and reliable compensation. |
| Valence | Overall job satisfaction | Overall job satisfaction demonstrates the value workers place on timely payments and financial stability, enhancing the attractiveness of achieving performance goals. |
|  | Prevalence of cash payments vs. digital payments | The higher prevalence of digital payments over cash payments over cash payments indicates a preference for more reliable and efficient compensation methods, making performance goals more desirable and valued by workers. |
