# Supplementary material for: Digital payments of health workers within vaccination campaigns: a mixed-methods study in Chad
Source: BMJ Glob Health. 2026 Jun 24;11(6):e018989. doi: 10.1136/bmjgh-2025-018989 (PMC13295920; doi:10.1136/bmjgh-2025-018989)
Supplement: online supplemental table 4 [file bmjgh-11-6-s009.docx]

**Supplementary table 4:** Qualitative insights mapped into the Expectancy Theory.

| **Expectancy theory** | **Theme** | **Sub-theme** | **Qualitative insight** |
| --- | --- | --- | --- |
| Expectancy | Payment set-up | Utilization | Training and system familiarity increase belief that effort improves performance. |
|  |  | Implementation | Involvement in implementation enhances belief in system effectiveness. |
|  |  | Responsibility | Collaborative approach reinforces belief in control over outcomes. |
| Instrumentality | Acceptance of the digital payment system | Job improvement | Digital payments improve job processes, linking good performance to rewards. |
|  |  | Readiness of vaccine workers | Mixed readiness among workers affects belief in reward reliability. |
|  |  | Preference for cash payments | Concerns about efficiency and reliability of digital payments. |
|  | Health system improvements | Efficiency | Increased payment speed and reduced delays strengthen the link between performance and rewards. |
|  |  | Transparency | Direct payments to workers reduce complaints and enhance perceived reward reliability. |
|  |  | Human resources | Improved management of resources and reduced for physical travel. |
| Valence |  | Experiences of vaccine workers | Direct payment into accounts increases the perceived value of rewards. |
|  | Challenges in the implementation and use of the digital payment system | Supplemental fees | Concerns about withdrawal fees reduce the perceived value of the rewards. |
|  |  | Technical issues | Technical challenges impact the perceived value and effectiveness of digital payments. |
|  |  | Slow payments | Delays in payment negatively affect workers’ perception of reward reliability and value. |
|  |  | Disagreement over distribution of money | Conflicts over fund allocation impact trust and perceived fairness in the distribution of rewards. |
