# Supplementary material for: Digital payments of health workers within vaccination campaigns: a mixed-methods study in Chad
Source: BMJ Glob Health. 2026 Jun 24;11(6):e018989. doi: 10.1136/bmjgh-2025-018989 (PMC13295920; doi:10.1136/bmjgh-2025-018989)
Supplement: online supplemental table 5 [file bmjgh-11-6-s010.docx]

**Supplementary table 5:** Characteristics of selected health facilities.

|  | **Comparison provinces (n = 662)** | **Mobile money implementing provinces (n = 848)** |
| --- | --- | --- |
|  | **Count (%)** | |
| ***Health workers working in health facilities*** | | |
| **Number of facilities** | 332 | 425 |
| **Number of provinces** | 5 (41.67) | 7 (58.33) |
| **Facility ownership** |  |  |
| Public | 567 (85.65) | 714 (84.2) |
| Private | 73 (11.03) | 88 (10.38) |
| Religious | 22 (3.32) | 46 (5.42) |
| **Facility location** |  |  |
| Rural | 564 (85.2) | 597 (70.4) |
| Urban | 98 (14.8) | 251 (29.6) |
| **Safety** |  |  |
| Safe zone | 519 (78.4) | 731 (86.2) |
| Compromised | 143 (21.6) | 117 (13.8) |
| **Accountant in facility** |  |  |
| Yes | 37 (5.59) | 105 (12.38) |
| No | 625 (94.41) | 743 (87.62) |
