# Supplementary material for: Digital payments of health workers within vaccination campaigns: a mixed-methods study in Chad
Source: BMJ Glob Health. 2026 Jun 24;11(6):e018989. doi: 10.1136/bmjgh-2025-018989 (PMC13295920; doi:10.1136/bmjgh-2025-018989)
Supplement: online supplemental table 6 [file bmjgh-11-6-s011.docx]

**Supplementary table 6:** Sample characteristics (health workers) stratified by health workers versus facility managers.

|  | | **Comparison districts (n = 662)** | | | **Mobile money implementing districts (n = 848)** | | |
| --- | --- | --- | --- | --- | --- | --- | --- |
|  | | **Managers (n = 328)** | **Vaccination workers (n = 334)** | **Total (n = 662)** | **Managers (n = 386)** | **Vaccination workers (n = 462)** | **Total (n = 848)** |
| **Education** | |  |  |  |  |  |  |
|  | None | 0 (0) | 1 (0.30) | 1 (0.15) | 1 (0.60) | 12 (2.60) | 13 (1.53) |
|  | Primary | 0 (0) | 34 (10.18) | 34 (5.14) | 3 (0.78) | 41 (8.87) | 44 (5.19) |
|  | Secondary | 69 (21.04) | 245 (73.35) | 314 (47.43) | 60 (15.54) | 254 (54.98) | 314 (37.03) |
|  | Higher | 259 (78.96) | 54 (16.17) | 313 (47.28) | 322 (83.42) | 155 (33.55) | 477 (56.25) |
| **Contract type** | |  |  |  |  |  |  |
|  | No contract | 84 (25.61) | 248 (74.25) | 332 (50.15) | 71 (18.4) | 236 (51.08) | 307 (36.2) |
|  | Short term | 19 (5.79) | 27 (8.08) | 46 (6.95) | 10 (2.59) | 31 (6.71) | 41 (4.83) |
|  | Fixed term | 89 (27.13) | 45 (13.47) | 134 (20.24) | 61 (15.80) | 41 (8.87) | 102 (12.03) |
|  | Permanent | 136 (41.46) | 14 (4.19) | 150 (22.66) | 244 (63.21) | 154 (33.33) | 398 (46.93) |
| **Cadre** | |  |  |  |  |  |  |
|  | Nurse | 144 (43.90) | 97 (29.04) | 241 (36.40) | 239 (61.92) | 194 (41.99) | 433 (51.06) |
|  | Midwife | 7 (2.13) | 27 (8.08) | 34 (5.14) | 23 (5.96) | 26 (5.63) | 49 (5.78) |
|  | Doctor | 2 (0.61) | 4 (1.20) | 6 (0.91) | 0 (0) | 0 (0) | 0 (0) |
|  | Manager | 145 (44.21) | 5 (1.50) | 150 (22.66) | 40 (10.36) | 10 (2.16) | 50 (5.90) |
|  | Others | 30 (9.15) | 201 (60.18) | 231 (34.89) | 84 (21.76) | 232 (50.22) | 316 (37.26) |
| **Sufficient funding** | |  |  |  |  |  |  |
|  | Yes | 57 (17.38) | 45 (13.47) | 102 (15.41) | 102 (26.42) | 161 (34.85) | 263 (31.01) |
|  | No | 271 (82.62) | 289 (86.53) | 560 (84.59) | 284 (73.58) | 301 (65.15) | 585 (68.99) |
