# Supplementary material for: Digital payments of health workers within vaccination campaigns: a mixed-methods study in Chad
Source: BMJ Glob Health. 2026 Jun 24;11(6):e018989. doi: 10.1136/bmjgh-2025-018989 (PMC13295920; doi:10.1136/bmjgh-2025-018989)
Supplement: online supplemental table 7 [file bmjgh-11-6-s012.docx]

**Supplementary table 7:** Payment experiences and confidence in digital payments among health facility managers, by province type.

|  |  | **Comparison provinces *(n = 328)*** | **Mobile money implementing provinces *(n = 386)*** | **Total**  ***(n = 714)*** |
| --- | --- | --- | --- | --- |
|  |  | **Count (%)** | | |
| **Confidence in digital payment system: if need cash out of digital payment** | | | | |
|  | Not very confident or not all confident | 68 (20.73) | 46 (11.92) | 114 (15.97) |
|  | Somewhat confident or very confident | 250 (76.22) | 323 (83.68) | 573 (80.25) |
|  | Do not know | 10 (3.05) | 17 (4.40) | 27 (3.78) |
| **Number of times received digital payments in the last 3 months** | | | | |
|  | None | 101 (30.79) | 125 (32.38) | 226 (31.65) |
|  | Once | 105 (18.9) | 59 (15.28) | 164 (22.97) |
|  | 2-3 times | 95 (28.96) | 192 (41.19) | 287 (40.20) |
|  | Missing | 27 (8.23) | 10 (2.59) | 37 (5.18) |
| **Number of times received cash payments in the last 3 months** | | | | |
|  | None | 23 (7.01) | 211 (54.66) | 234 (32.77) |
|  | Once | 62 (18.9) | 75 (19.43) | 137 (19.19) |
|  | 2-3 times | 199 (60.67) | 87 (22.54) | 286 (40.06) |
|  | Missing | 44 (13.41) | 13 (3.37) | 57 (7.98) |
| **Experienced payment delays** | | | | |
|  | Never | 87 (26.52) | 256 (66.32) | 343 (48.04) |
|  | Yes, less than half | 125 (38.11) | 73 (18.91) | 198 (27.73) |
|  | Yes, more than half | 116 (35.37) | 57 (14.77) | 173 (24.23) |
