# Supplementary material for: Digital payments of health workers within vaccination campaigns: a mixed-methods study in Chad
Source: BMJ Glob Health. 2026 Jun 24;11(6):e018989. doi: 10.1136/bmjgh-2025-018989 (PMC13295920; doi:10.1136/bmjgh-2025-018989)
Supplement: online supplemental table 8 [file bmjgh-11-6-s013.docx]

**Supplementary table 8:** Full multivariable OLS regression outputs corresponding to Table 3.

**Outcome: Work motivation**

**Panel A: Managers**

|  | **Full sample** | **Provinces without PBF** | **Provinces with PBF** |
| --- | --- | --- | --- |
| **Digital payment (vs. cash)** | 0.53^***^  (0.35, 0.70) | -0.26  (-0.53, 0.02) | 1.21^***^  (1.00, 1.43) |
| PBF | 0.50^***^  (0.33, 0.67) | - | - |
| Age (years) | 0.01  (-0.001, 0.02) | 0.01  (-0.004, 0.03) | 0.01  (-0.01, 0.02) |
| Female (ref: male) | -0.08  (-0.31, 0.15) | -0.34^*^  (-0.66, -0.03) | 0.04  (-0.24, 0.32) |
| Cadre | -0.07  (-0.15, 0.01) | 0.13^*^  (0.03, 0.24) | -0.28^***^  (-0.38, -0.17) |
| Contract type | -0.02  (-0.08, 0.05) | -0.03  (-0.11, 0.06) | -0.18^***^  (-0.27, -0.08) |
| Location | 0.36^***^  (0.16, 0.56) | 0.65^***^  (0.37, 0.92) | -0.004  (-0.32, 0.31) |
| Safety | 0.24^*^  (0.001, 0.46) | -0.005  (-0.25, 0.24) | 0.55  (-0.0001, 1.11) |
| Education | -0.06  (-0.13, 0.01) | -0.08  (-0.16, 0.01) | -0.12^*^  (-0.23, -0.02) |
| Population size (per 10,000) | 0.02  (-0.03, 0.08) | 0.03  (-0.09, 0.16) | 0.03  (-0.02, 0.07) |
| Constant | 2.18  (1.29, 3.06) | 1.94^**^  (0.76, 3.12) | 4.23^***^  (3.03, 5.42) |
| N | 714 | 374 | 340 |

**Notes:** Coefficients with 95% confidence intervals are presented from OLS regression models. Statistical significance is indicated by stars, with *p < 0.05, **p < 0.01, ***p < 0.001. All models were adjusted for potential confounders including age, sex, cadre, education, contract type, location, safety, and population size. This table includes complete model specifications and coefficient estimate for all confounders. Results are shown for the full sample, as well as separately for provinces without PBF and provinces with PBF exposure. Panel A presents results for managers, and Panel B for non-managers. The outcomes were measured on 5-point Likert scales, with work motivation ranging from 1 (very low) to 5 (very high), and payment and job satisfaction ranging from 1 (very dissatisfied) to 5 (very satisfied). Higher coefficients indicate a relative increase in the outcome score, measured in Likert points, associated with the mobile money intervention.

**Panel B: Non-managers**

|  | **Full sample** | **Provinces without PBF** | **Provinces with PBF** |
| --- | --- | --- | --- |
| **Digital payment (vs. cash)** | -0.02  (-0.18, 0.14) | 0.14  (-0.13, 0.41) | -0.41^**^  (-0.66, -0.15) |
| PBF | 0.03  (-0.13, 0.18) | - | - |
| Age (years) | -0.01  (-0.02, 0.004) | -0.01  (-0.02, 0.01) | -0.002  (-0.016, 0.012) |
| Female (ref: male) | -0.18  (-0.40, 0.03) | -0.30  (-0.61, 0.003) | 0.22  (-0.13, 0.58) |
| Cadre | 0.11  (-0.0003, 0.21) | 0.003  (-0.14, 0.15) | 0.03  (-0.14, 0.20) |
| Contract type | 0.01  (-0.05, 0.08) | -0.12^*^  (-0.22, -0.02) | 0.12^*^  (0.03, 0.22) |
| Location | -0.003  (-0.19, 0.19) | 0.47^**^  (0.20, 0.74) | -0.45^***^  (-0.69, -0.20) |
| Safety | 0.14  (-0.04, 0.33) | 0.37^**^  (0.13, 0.61) | -0.04  (-0.37, 0.30) |
| Education | -0.18^***^  (-0.24, -0.12) | -0.07  (-0.16, 0.02) | -0.18^***^  (-0.26, -0.10) |
| Population size (per 10,000) | 0.03  (-0.04, 0.10) | 0.12^*^  (0.00, 0.24) | -0.02  (-0.08, 0.04) |
| Constant | 3.31  (2.42, 4.20) | 2.78^***^  (1.55, 4.00) | 3.69^***^  (2.42, 4.95) |
| N | 796 | 372 | 424 |

**Notes:** Coefficients with 95% confidence intervals are presented from OLS regression models. Statistical significance is indicated by stars, with *p < 0.05, **p < 0.01, ***p < 0.001. All models were adjusted for potential confounders including age, sex, cadre, education, contract type, location, safety, and population size. This table includes complete model specifications and coefficient estimate for all confounders. Results are shown for the full sample, as well as separately for provinces without PBF and provinces with PBF exposure. Panel A presents results for managers, and Panel B for non-managers. The outcomes were measured on 5-point Likert scales, with work motivation ranging from 1 (very low) to 5 (very high), and payment and job satisfaction ranging from 1 (very dissatisfied) to 5 (very satisfied). Higher coefficients indicate a relative increase in the outcome score, measured in Likert points, associated with the mobile money intervention.

**Outcome: Payment satisfaction**

**Panel A: Managers**

|  | **Full sample** | **Provinces without PBF** | **Provinces with PBF** |
| --- | --- | --- | --- |
| **Digital payment (vs. cash)** | -0.05  (-0.24, 0.14) | -0.59^***^  (-0.86, -0.32) | 0.57^***^  (0.30, 0.83) |
| PBF | 0.35^***^  (0.17, 0.52) | - | - |
| Age (years) | 0.0002  (-0.009, 0.01) | -0.001  (-0.014, 0.01) | 0.003  (-0.01, 0.02) |
| Female (ref: male) | -0.06  (-0.26, 0.13) | -0.22  (-0.48, 0.05) | -0.06  (-0.34, 0.22) |
| Cadre | 0.04  (-0.04, 0.11) | 0.17^**^  (0.06, 0.28) | -0.09  (-0.19, 0.002) |
| Contract type | -0.003  (-0.07, 0.06) | -0.002  (-0.09, 0.08) | -0.10^*^  (-0.20, -0.001) |
| Location | 0.07  (-0.09, 0.24) | 0.09  (-0.14, 0.31) | 0.24  (-0.00012, 0.48) |
| Safety | 0.39  (0.16, 0.61) | 0.22  (-0.03, 0.46) | 0.55  (-0.09, 1.19) |
| Education | 0.02  (-0.04, 0.08) | 0.09  (-0.002. 0.17) | -0.15^***^  (-0.23, -0.07) |
| Population size (per 10,000) | 0.07  (0.02, 0.13) | 0.12  (0.02, 0.22) | 0.02  (-0.06, 0.10) |
| Constant | 1.51  (0.77, 2.26) | 1.34^**^  (0.39, 2.29) | 2.73^***^  (1.55, 3.90) |
| N | 714 | 374 | 340 |

**Notes:** Coefficients with 95% confidence intervals are presented from OLS regression models. Statistical significance is indicated by stars, with *p < 0.05, **p < 0.01, ***p < 0.001. All models were adjusted for potential confounders including age, sex, cadre, education, contract type, location, safety, and population size. This table includes complete model specifications and coefficient estimate for all confounders. Results are shown for the full sample, as well as separately for provinces without PBF and provinces with PBF exposure. Panel A presents results for managers, and Panel B for non-managers. The outcomes were measured on 5-point Likert scales, with work motivation ranging from 1 (very low) to 5 (very high), and payment and job satisfaction ranging from 1 (very dissatisfied) to 5 (very satisfied). Higher coefficients indicate a relative increase in the outcome score, measured in Likert points, associated with the mobile money intervention.

**Panel B: Non-managers**

|  | **Full sample** | **Provinces without PBF** | **Provinces with PBF** |
| --- | --- | --- | --- |
| **Digital payment (vs. cash)** | -0.25^***^  (-0.38, -0.11) | -0.22  (-0.49, 0.05) | -0.31^**^  (-0.51, -0.11) |
| PBF | 0.04  (-0.09, 0.17) | - | - |
| Age (years) | 0.01^*^  (0.001, 0.02) | 0.02^**^  (0.01, 0.03) | -0.003  (-0.0005, 0.58) |
| Female (ref: male) | 0.19^*^  (0.02, 0.37) | 0.10  (-0.14, 0.35) | 0.29  (-0.01, 0.01) |
| Cadre | 0.05  (-0.04, 0.14) | 0.02  (-0.12, 0.16) | 0.08  (-0.08, 0.24) |
| Contract type | 0.03  (-0.03, 0.09) | -0.04  (-0.12, 0.16) | 0.09^*^  (0.02, 0.16) |
| Location | 0.02  (-0.13, 0.17) | -0.08  (-0.31, 0.15) | 0.07  (-0.11, 0.26) |
| Safety | 0.014  (-0.14, 0.17) | -0.02  (-0.23, 0.20) | 0.10  (-0.15, 0.35) |
| Education | -0.01  (-0.06, 0.04) | -0.04  (-0.12, 0.04) | 0.01  (-0.06, 0.08) |
| Population size (per 10,000) | -0.03  (-0.08, 0.02) | -0.10^*^  (-0.19, -0.01) | 0.01  (-0.06, 0.07) |
| Constant | 1.46  (0.70, 2.22) | 1.99^***^  (0.88, 3.10) | 1.19^*^  (0.12, 2.26) |
| N | 796 | 372 | 424 |

**Notes:** Coefficients with 95% confidence intervals are presented from OLS regression models. Statistical significance is indicated by stars, with *p < 0.05, **p < 0.01, ***p < 0.001. All models were adjusted for potential confounders including age, sex, cadre, education, contract type, location, safety, and population size. This table includes complete model specifications and coefficient estimate for all confounders. Results are shown for the full sample, as well as separately for provinces without PBF and provinces with PBF exposure. Panel A presents results for managers, and Panel B for non-managers. The outcomes were measured on 5-point Likert scales, with work motivation ranging from 1 (very low) to 5 (very high), and payment and job satisfaction ranging from 1 (very dissatisfied) to 5 (very satisfied). Higher coefficients indicate a relative increase in the outcome score, measured in Likert points, associated with the mobile money intervention.

**Outcome: Job satisfaction**

**Panel A: Managers**

|  | **Full sample** | **Provinces without PBF** | **Provinces with PBF** |
| --- | --- | --- | --- |
| **Digital payment (vs. cash)** | 0.36^***^  (0.16, 0.55) | -0.31^*^  (-0.60, -0.03) | 0.90^***^  (0.64, 1.16) |
| PBF | 0.17  (-0.01, 0.34) | - | - |
| Age (years) | 0.01  (-0.01, 0.02) | 0.01  (-0.01, 0.03) | -0.001  (-0.01, 0.01) |
| Female (ref: male) | -0.02  (-0.27, 0.22) | -0.30  (-0.66, 0.07) | 0.11  (-0.22, 0.44) |
| Cadre | 0.04  (-0.04, 0.12) | 0.15^*^  (0.03, 0.27) | -0.03  (-0.12, 0.05) |
| Contract type | 0.005  (-0.06, 0.07) | -0.06  (-0.15, 0.03) | -0.08^*^  (-0.15, -0.002) |
| Location | 0.21^*^  (0.01, 0.41) | 0.38^*^  (0.10, 0.65) | 0.11  (-0.15, 0.37) |
| Safety | 0.31^*^  (0.07, 0.55) | 0.21  (-0.06, 0.48) | 0.08  (-0.37, 0.52) |
| Education | 0.01  (-0.05, 0.07) | -0.02  (-0.11, 0.06) | -0.03  (-0.11, 0.05) |
| Population size (per 10,000) | 0.08  (0.02, 0.14) | 0.09  (-0.04, 0.21) | 0.06^*^  (0.01, 0.12) |
| Constant | 1.01^*^  (0.18, 1.84) | 1.33^*^  (0.20, 2.46) | 2.06^**^  (0.87, 3.25) |
| N | 714 | 374 | 340 |

**Notes:** Coefficients with 95% confidence intervals are presented from OLS regression models. Statistical significance is indicated by stars, with *p < 0.05, **p < 0.01, ***p < 0.001. All models were adjusted for potential confounders including age, sex, cadre, education, contract type, location, safety, and population size. This table includes complete model specifications and coefficient estimate for all confounders. Results are shown for the full sample, as well as separately for provinces without PBF and provinces with PBF exposure. Panel A presents results for managers, and Panel B for non-managers. The outcomes were measured on 5-point Likert scales, with work motivation ranging from 1 (very low) to 5 (very high), and payment and job satisfaction ranging from 1 (very dissatisfied) to 5 (very satisfied). Higher coefficients indicate a relative increase in the outcome score, measured in Likert points, associated with the mobile money intervention.

**Panel B: Non-managers**

|  | **Full sample** | **Provinces without PBF** | **Provinces with PBF** |
| --- | --- | --- | --- |
| **Digital payment (vs. cash)** | -0.05  (-0.17, 0.08) | -0.29^*^  (-0.54, 0.04) | 0.01  (-0.17, 0.20) |
| PBF | -0.08  (-0.20, 0.04) | - | - |
| Age (years) | -0.004  (-0.01, 0.004) | -0.01^*^  (-0.02, -0.001) | 0.01  (-0.01. 0.02) |
| Female (ref: male) | -0.01  (-0.21, 0.19) | -0.25^*^  (-0.55, 0.04) | 0.18  (-0.09, 0.46) |
| Cadre | -0.05  (-0.15, 0.05) | -0.04  (-0.19, 0.12) | -0.05  (-0.21, 0.11) |
| Contract type | 0.10^***^  (0.05, 0.16) | -0.05  (-0.15, 0.04) | 0.13^**^  (0.05, 0.21) |
| Location | 0.01  (-0.12, 0.15) | 0.16  (-0.07, 0.38) | -0.06  (-0.22, 0.10) |
| Safety | 0.05  (-0.11, 0.20) | 0.20  (-0.04, 0.43) | -0.26^**^  (-0.45, -0.07) |
| Education | -0.02  (-0.07, 0.03) | 0.03  (-0.05, -0.001) | -0.05  (-0.11, 0.01) |
| Population size (per 10,000) | -0.02  (-0.07, 0.03) | -0.05  (-0.16, 0.05) | 0.00  (-0.05, 0.05) |
| Constant | 2.80  (2.01, 3.59) | 3.20^***^  (2.07, 4.32) | 2.47^***^  (1.42, 3.52) |
| N | 796 | 372 | 424 |

**Notes:** Coefficients with 95% confidence intervals are presented from OLS regression models. Statistical significance is indicated by stars, with *p < 0.05, **p < 0.01, ***p < 0.001. All models were adjusted for potential confounders including age, sex, cadre, education, contract type, location, safety, and population size. This table includes complete model specifications and coefficient estimate for all confounders. Results are shown for the full sample, as well as separately for provinces without PBF and provinces with PBF exposure. Panel A presents results for managers, and Panel B for non-managers. The outcomes were measured on 5-point Likert scales, with work motivation ranging from 1 (very low) to 5 (very high), and payment and job satisfaction ranging from 1 (very dissatisfied) to 5 (very satisfied). Higher coefficients indicate a relative increase in the outcome score, measured in Likert points, associated with the mobile money intervention.
