# Supplementary material for: Digital payments of health workers within vaccination campaigns: a mixed-methods study in Chad
Source: BMJ Glob Health. 2026 Jun 24;11(6):e018989. doi: 10.1136/bmjgh-2025-018989 (PMC13295920; doi:10.1136/bmjgh-2025-018989)
Supplement: online supplemental table 9 [file bmjgh-11-6-s014.docx]

**Supplementary table 9:** Distribution of motivation and satisfaction among health workers, by province type and sex.

| **Outcome**  **(5-point Likert scale)** | **Comparison provinces (n = 662)** | **Mobile money implementing provinces (n = 848)** | **Total**  **(n = 1,510)** |
| --- | --- | --- | --- |
|  | **Count (%)** | | |
| **Panel A: Female health workers (n = 212)** | | | |
| **Work motivation** | | | |
| Strongly disagree | 0 (0.00) | 0 (0.00) | 0 (0.00) |
| Somewhat disagree | 0 (0.00) | 0 (0.00) | 0 (0.00) |
| Neither agree nor disagree | 1 (1.14) | 7 (5.65) | 8 (3.77) |
| Somewhat agree | 53 (60.23) | 34 (27.42) | 87 (41.04) |
| Strongly agree | 34 (38.64) | 83 (66.94) | 117 (55.19) |
| **Payment satisfaction** | | | |
| Strongly disagree | 4 (4.55) | 5 (4.03) | 9 (4.25) |
| Somewhat disagree | 22 (25.00) | 16 (12.90) | 38 (17.92) |
| Neither agree nor disagree | 3 (3.41) | 17 (13.71) | 20 (9.43) |
| Somewhat agree | 57 (64.77) | 77 (62.10) | 134 (63.21) |
| Strongly agree | 2 (2.27) | 9 (7.26) | 11 (5.19) |
| **Overall job satisfaction** | | | |
| Strongly disagree | 3 (3.41) | 9 (7.26) | 12 (5.66) |
| Somewhat disagree | 2 (2.27) | 5 (4.03) | 7 (3.30) |
| Neither agree nor disagree | 5 (5.68) | 11 (8.87) | 16 (7.55) |
| Somewhat agree | 65 (73.86) | 77 (62.10) | 142 (66.98) |
| Strongly agree | 13 (14.77) | 22 (17.74) | 35 (16.51) |
| **Panel B: Male health workers (n = 1298)** | | | |
| **Work motivation** | | | |
| Strongly disagree | 3 (0.52) | 3 (0.41) | 6 (0.46) |
| Somewhat disagree | 12 (2.09) | 10 (1.38) | 22 (1.69) |
| Neither agree nor disagree | 25 (4.36) | 38 (5.25) | 63 (4.85) |
| Somewhat agree | 312 (54.36) | 326 (45.03) | 638 (49.15) |
| Strongly agree | 222 (38.68) | 347 (47.93) | 569 (43.84) |
| **Payment satisfaction** | | | |
| Strongly disagree | 36 (6.27) | 18 (2.49) | 54 (4.16) |
| Somewhat disagree | 197 (32.32) | 149 (20.58) | 346 (26.66) |
| Neither agree nor disagree | 43 (7.49) | 121 (16.71) | 164 (12.63) |
| Somewhat agree | 283 (49.30) | 354 (48.90) | 637 (49.08) |
| Strongly agree | 15 (2.61) | 82 (11.33) | 97 (7.47) |
| **Overall job satisfaction** | | | |
| Strongly disagree | 16 (2.79) | 17 (2.35) | 33 (2.54) |
| Somewhat disagree | 17 (2.96) | 63 (8.70) | 80 (6.16) |
| Neither agree nor disagree | 12 (2.09) | 68 (9.39) | 80 (6.16) |
| Somewhat agree | 476 (82.93) | 428 (59.12) | 904 (69.65) |
| Strongly agree | 53 (9.23) | 148 (20.44) | 201 (15.49) |
