# Supplementary material for: Digital payments of health workers within vaccination campaigns: a mixed-methods study in Chad
Source: BMJ Glob Health. 2026 Jun 24;11(6):e018989. doi: 10.1136/bmjgh-2025-018989 (PMC13295920; doi:10.1136/bmjgh-2025-018989)
Supplement: online supplemental table 10 [file bmjgh-11-6-s015.docx]

**Supplementary table 10:** Subgroup analysis (ordinal) of the effect of mobile money intervention on health worker outcomes, by sex.

| **Outcome** | **Full sample** | **Provinces without PBF** | **Provinces with PBF** |
| --- | --- | --- | --- |
| **Panel A: Female health workers** | | | |
| Work motivation | 0.26  (-0.11, 0.63) | -0.06  (-0.74, 0.62) | 0.48^*^  (0.01, 0.94) |
| Payment satisfaction | -0.02  (-0.35, 0.31) | -0.09  (-0.63, 0.46) | 0.12  (-0.27, 0.51) |
| Job satisfaction | -0.03  (-0.43, 0.37) | -0.25  (-1.04, 0.53) | 0.23  (-0.29, 0.76) |
| N | 212 | 119 | 93 |
| **Panel B: Male health workers** | | | |
| Work motivation | 0.15^*^  (0.01, 0.29) | 0.0009  (-0.23, 0.23) | 0.17  (-0.04, 0.38) |
| Payment satisfaction | -0.19^**^  (-0.31, -0.07) | -0.44^***^  (-0.65, -0.3) | 0.02  (-0.16, 0.19) |
| Job satisfaction | 0.13^*^  (0.01, 0.25) | -0.32^**^  (-0.53, -0.11) | 0.45^***^  (0.28, 0.62) |
| N | 1298 | 627 | 671 |

**Notes:** Coefficients with 95% confidence intervals are presented from OLS regression models. Statistical significance is indicated by stars, with *p < 0.05, **p < 0.01, ***p < 0.001. Subgroup analyses were conducted separately for female health workers (Panel A) and male health workers (Panel B), in line with SAGER recommendations. All models adjust for potential confounders, including age, sex, cadre, education, contract type, location, safety, and population size. Results are presented for the full sample, and separately for provinces without PBF and provinces with PBF exposure.
