# Supplementary material for: Digital payments of health workers within vaccination campaigns: a mixed-methods study in Chad
Source: BMJ Glob Health. 2026 Jun 24;11(6):e018989. doi: 10.1136/bmjgh-2025-018989 (PMC13295920; doi:10.1136/bmjgh-2025-018989)
Supplement: online supplemental table 11 [file bmjgh-11-6-s016.docx]

**Supplementary table 11:** Distribution of motivation and satisfaction outcomes under alternative binary definitions.

| **Outcome** | **Overall**  **(N = 1510)** | **Mobile money implementing provinces**  **(N = 848)** | **Control provinces**  **(N = 662)** |
| --- | --- | --- | --- |
|  | **Count (%)** | | |
| **Panel A: Binary cut-off 1** | | | |
| Work motivation |  |  |  |
| Not very motivated | 824 (54.6) | 406 (61.3) | 418 (49.3) |
| Very motivated | 686 (45.4) | 256 (38.7) | 430 (50.7) |
| Payment satisfaction |  |  |  |
| Not very satisfied | 1402 (92.9) | 645 (97.4) | 757 (89.3) |
| Very satisfied | 108 (7.1) | 17 (2.6) | 91 (10.7) |
| Job satisfaction |  |  |  |
| Not very satisfied | 1274 (84.3) | 596 (90.0) | 678 (79.9) |
| Very satisfied | 236 (15.7) | 66 (10.0) | 170 (20.1) |
| **Panel B: Binary cut-off 2** | | | |
| Work motivation |  |  |  |
| Not motivated | 99 (6.6) | 58 (6.8) | 41 (6.2) |
| Motivated or very motivated | 1411 (93.4) | 790 (93.2) | 621 (93.8) |
| Payment satisfaction |  |  |  |
| Not satisfied | 631 (41.8) | 326 (38.4) | 305 (46.1) |
| Satisfied or very satisfied | 879 (58.2) | 522 (61.6) | 357 (53.9) |
| Job satisfaction |  |  |  |
| Not satisfied | 228 (15.1) | 173 (20.4) | 55 (8.3) |
| Satisfied or very satisfied | 1282 (84.9) | 675 (79.6) | 607 (91.7) |

**Notes:** Cut-off 1 collapses the Likert scale into “very motivated/satisfied” vs. all other categories; Cut-off 2 collapses the scale into “motivated or very motivated/satisfied” vs. low categories; Values are presented as n (%).
