# Supplementary material for: Digital payments of health workers within vaccination campaigns: a mixed-methods study in Chad
Source: BMJ Glob Health. 2026 Jun 24;11(6):e018989. doi: 10.1136/bmjgh-2025-018989 (PMC13295920; doi:10.1136/bmjgh-2025-018989)
Supplement: online supplemental table 12 [file bmjgh-11-6-s017.docx]

**Supplementary table 12:** Effects of mobile money on health worker outcomes across alternative regression specifications (binary cut-off 1 only).

|  | **Full sample** | **Provinces without PBF** | **Provinces with PBF** |
| --- | --- | --- | --- |
| **Panel A: Managers** | | | |
| Work motivation | 0.29^***^  (0.20, 0.37) | -0.05  (-0.17, 0.08) | 0.60^***^  (0.50, 0.70) |
| Payment satisfaction | 0.19^***^  (0.14, 0.23) | 0.07^**^  (0.02, 0.11) | 0.34^***^  (0.25, 0.42) |
| Job satisfaction | 0.23^***^  (0.17, 0.29) | 0.04  (-0.04, 0.12) | 0.41^***^  (0.32, 0.50) |
| N | 714 | 374 | 340 |
| **Panel B: Non-managers** | | | |
| Work motivation | -0.15  (-0.09, 0.06) | 0.04  (-0.07, 0.16) | -0.16^**^  (-0.27, -0.05) |
| Payment satisfaction | 0.03  (0.00, 0.06) | 0.03  (-0.02, 0.07) | 0.02  (-0.04, 0.07) |
| Job satisfaction | -0.01  (-0.06, 0.04) | 0.01  (-0.08, 0.096) | -0.02  (-0.08, 0.04) |
| N | 796 | 372 | 424 |

**Notes:** Coefficients with 95% confidence intervals are presented from OLS regression models. Statistical significance is indicated by stars, with *p < 0.05, **p < 0.01, ***p < 0.001. All models were adjusted for potential confounders including age, sex, cadre, education, contract type, location, safety, and population size. Results are shown for the full sample, as well as separately for provinces without PBF and provinces with PBF exposure. Panel A presents results for managers, and Panel B for non-managers. Outcomes are defined using a binary cut-off at the highest category only (very motivated/satisfied = 1 vs. all others = 0).
