# Supplementary material for: Digital payments of health workers within vaccination campaigns: a mixed-methods study in Chad
Source: BMJ Glob Health. 2026 Jun 24;11(6):e018989. doi: 10.1136/bmjgh-2025-018989 (PMC13295920; doi:10.1136/bmjgh-2025-018989)
Supplement: online supplemental table 14 [file bmjgh-11-6-s020.docx]

**Supplementary table 14:** Joint display of integrated quantitative and qualitative findings on the effects of mobile money payments on health worker motivation and satisfaction in Chad.

| **Outcome** | **Quantitative results** | **Qualitative findings** | **Integration** |
| --- | --- | --- | --- |
| **Expectancy** | | | |
| Work motivation | - Managers in PBF provinces have higher mean motivation scores; managers in non-PBF show no clear gains. - Non-managers in PBF show lower motivation; effects are small or non-significant for non-managers in non-PBF. | - Managers described efficiency gains: “Digital payment are currently used in all centers in my area of responsibility.” - Others acting as vaccinators highlighted burdens, including fees, distance, network. | - Convergence: Higher motivation for managers. - Divergence explained by role overlap and semi-direct model where managers also bore frontline burdens when cashing out. |
| Work attendance | - Missed workdays are lower in mobile money provinces (23% vs. 34%). | - Staff reported that digital payments reduced travel time to collect funds, allowing them to attend work consistently. | - Convergence: Quantitative reductions in absence supported by qualitative accounts of time saved. |
| Confidence in digital payment systems | - Managers only: Most (80%) report being somewhat/very confident in digital payments. | - Some vaccinators initially (“we thought it was a scam”) mistrusted the system but later acknowledged greater reliability. | - Convergent overall, with divergence where low digital literacy or poor rollout undermined early confidence. |
| **Instrumentality** | | | |
| Payment satisfaction | - Managers in PBF report higher mean satisfaction; managers in non-PBF report lower satisfaction. - Non-managers show lower satisfaction in PBF and a negative or non-significant pattern in non-PBF. | - Positive: “The waiting line has changed, directly into the beneficiary account.” - Negative: “You have to travel 58km” / “Fees reduced the amount I receive.” | - Convergence: Transparency improved satisfaction for some, but barriers such as fees and distance reduced satisfaction, explaining mixed quantitative results. |
| Positive payment experience | - Higher proportion of workers in mobile money provinces rate payment experience as “good/very good.” | - Workers cited speed, security, and transparency as major advantages of mobile payments. | - Convergence: Both strands highlight improved payment experience. |
| Reduced payment delays | - Fewer delays in mobile money provinces (34% vs. 74%). | - Respondents noted “payments arrive directly without waiting for managers [district officials]”. | - Convergence: Improved timeliness confirmed across strands. |
| Frequency of digital payments | - Most managers in mobile money provinces reported multiple digital payments in 3 months. | - Participants described payments becoming routine and predictable. | - Convergent: Frequency supported by qualitative perceptions of reliability. |
| **Valence** | | | |
| Overall job satisfaction | - Higher among managers in PBF provinces. - Non-managers in PBF show no clear difference. - Lower among non-managers in non-PBF. | - “Security is guaranteed because everyone has their money with them.” - But hybrid manager-vaccinator roles noted, “System is not effective for our area.” | - Convergent: Managers valued reliability, but job satisfaction tempered where cash-out responsibilities or barriers persisted. |
| Prevalence of digital vs. cash payments | - Digital payments more prevalent in mobile money provinces. | - Workers appreciated digital transfers but noted cash was still used to pay vaccinators under the semi-direct model, with related burdens (travel, fees). | - Convergent: Preference shifting toward digital, but divergence in practice since cash remained necessary for redistribution under semi-direct arrangements. |
