# Supplementary material for: Digital payments of health workers within vaccination campaigns: a mixed-methods study in Chad
Source: BMJ Glob Health. 2026 Jun 24;11(6):e018989. doi: 10.1136/bmjgh-2025-018989 (PMC13295920; doi:10.1136/bmjgh-2025-018989)
Supplement: online supplemental file 7 [file bmjgh-11-6-s021.docx]

### BMJ Global Health Author Reflexivity Statement

Adapted from Morton, B., Vercueil, A., Masekela, R., Heinz, E., Reimer, L., Saleh, S., Kalinga, C., Seekles, M., Biccard, B., Chakaya, J., Abimbola, S., Obasi, A. and Oriyo, N. (2022), Consensus statement on measures to promote equitable authorship in the publication of research from international partnerships. Anaesthesia, 77: 264-276. <https://doi.org/10.1111/anae.15597>

| **Study conceptualisation** | |
| --- | --- |
| 1. How does this study address local research and policy priorities? | This study addresses a critical policy priority in Chad: improving the timeliness, transparency, and reliability of payments to health workers, particularly during vaccination campaigns. Delayed and inconsistent payments have long undermined workforce motivation and service delivery, yet evidence on how digital payment systems perform in fragile settings remains limited. By evaluating the real-world rollout of mobile money payments across 12 provinces, this mixed-methods study generates locally relevant evidence on both the benefits and implementation challenges of payment digitization. The findings provide actionable guidance for national policymakers and partners on how payment modality, rollout design, and alignment with performance-based financing shape health worker experiences. These insights are directly relevant to ongoing health financing, digital public financial management, and immunization system reforms in Chad and similar low-resource contexts. |
| 1. How were local researchers involved in study design? | Local researchers and practitioners in Chad were actively involved at all stages of the study design. The research questions were developed collaboratively with national stakeholders, including public health officials and implementing partners, to ensure alignment with local priorities and operational realities. Chadian researchers contributed to the adaptation of survey instruments and interview guides to ensure cultural relevance, appropriate language, and contextual sensitivity. Local team members also advised on sampling strategies, site selection, and the interpretation of findings, drawing on their in-depth knowledge of the health system and vaccination campaign operations. This collaborative approach ensured that the study design reflected local perspectives and addressed policy-relevant questions grounded in Chad’s health system context. |
| **Research management** | |
| 1. How has funding been used to support the local research team(s)? | Study funding was used to directly support local research teams in Chad through fair compensation for data collection, transcription, translation, and qualitative analysis activities. Local researchers and field staff were engaged as paid collaborators and received training in research ethics, mixed-methods data collection, and qualitative interviewing. Resources were also allocated to support local coordination, field logistics, and stakeholder engagement, including dissemination of findings to national partners. This approach ensured meaningful participation of local researchers, strengthened local research capacity, and promoted equitable collaboration throughout the study. |
| **Data acquisition and analysis** | |
| 1. How are research staff who conducted data collection acknowledged? | Research staff who conducted data collection are formally acknowledged through authorship where criteria were met and in the acknowledgements section of the manuscript for their substantive contributions. Field researchers, interviewers, and coordinators are recognized for their roles in data collection, transcription, translation, and contextual interpretation, in line with ethical authorship and acknowledgment guidelines. |
| 1. How have members of the research partnership been provided with access to study data? | Members of the research partnership were provided with access to study data through secure, password-protected data-sharing platforms, consistent with ethical approvals and confidentiality requirements. De-identified quantitative datasets, qualitative transcripts, and analysis outputs were shared with partner institutions to support joint analysis, interpretation, and manuscript development. |
| 1. How were data used to develop analytical skills within the partnership? | Study data were used as a practical training resource to build analytical capacity within the partnership. Local researchers were actively involved in data cleaning, coding of qualitative transcripts, and interpretation of quantitative results through iterative analysis meetings. This hands-on engagement strengthened skills in mixed-methods analysis, qualitative coding, and interpretation of policy-relevant findings, fostering long-term research capacity. |
| **Data interpretation** | |
| 1. How have research partners collaborated in interpreting study data? | Research partners jointly interpreted study data through iterative analysis discussions that integrated quantitative results with qualitative insights. Local partners contributed contextual knowledge to explain observed patterns, validate emerging themes, and identify implementation factors influencing results. This collaborative approach ensured interpretations were grounded in local health system realities and policy relevance. |
| **Drafting and revising for intellectual content** | |
| 1. How were research partners supported to develop writing skills? | Research partners were supported to develop writing skills through active involvement in manuscript drafting, iterative revisions, and structured feedback from senior investigators. Local researchers contributed to drafting sections of the manuscript, reviewing analyses, and responding to peer-review comments, gaining hands-on experience in scientific writing and publication processes. |
| 1. How will research products be shared to address local needs? | Research products will be shared with national stakeholders through policy briefs, stakeholder meetings, and presentations to the Ministry of Public Health and implementing partners. Findings will also be disseminated through local workshops and written summaries tailored to program managers to inform ongoing payment, immunization, and health financing reforms in Chad. |
| **Authorship** | |
| 1. How is the leadership, contribution and ownership of this work by LMIC researchers recognised within the authorship? | Leadership and ownership by LMIC researchers are reflected through first and senior authorship positions held by researchers based in Chad, as well as through substantive contributions to study design, data collection, analysis, and interpretation. Authorship decisions followed ICMJE criteria, ensuring that intellectual leadership and local expertise were appropriately recognized. |
| 1. How have early career researchers across the partnership been included within the authorship team? | Early career researchers were included as co-authors based on meaningful contributions to data collection, analysis, and manuscript development. They were actively engaged in drafting sections of the paper, reviewing analyses, and participating in revisions, providing opportunities for mentorship and professional development within the authorship team. |
| 1. How has gender balance been addressed within the authorship? | Gender balance was actively considered in forming the authorship team. Women researchers are represented among both senior and early career authors and contributed across key aspects of the study, including qualitative data collection, analysis, and interpretation. Efforts were made to ensure inclusive participation and recognition of contributions regardless of gender. |
| **Training** | |
| 1. How has the project contributed to training of LMIC researchers? | The project contributed to training LMIC researchers through hands-on involvement in mixed-methods study design, data collection, analysis, and manuscript preparation. Local researchers received training in research ethics, quantitative data management, qualitative interviewing and coding, and interpretation of findings, supported by mentorship from senior investigators. This experiential approach strengthened analytical, writing, and dissemination skills and supported longer-term research capacity within the partnership. |
| **Infrastructure** | |
| 1. How has the project contributed to improvements in local infrastructure? | The project contributed to improvements in local infrastructure by strengthening data collection and management systems used by local research teams, including secure digital data storage, standardized survey tools, and qualitative recording and transcription workflows. These resources remain available to local partners for future studies and program evaluations, supporting more efficient, ethical, and high-quality research in Chad. |
| **Governance** | |
| 1. What safeguarding procedures were used to protect local study participants and researchers? | Safeguarding procedures included approval from an institutional review board, comprehensive training of research staff in research ethics and safeguarding and obtaining informed consent from all participants. Data were de-identified and stored securely to protect confidentiality. Interviews were conducted in private, safe locations, and participation was voluntary with the right to withdraw at any time. Field teams followed established safety protocols, including risk assessments and supervisory support, to protect researchers working in fragile settings. |
